# *Lactobacillus reuteri* normalizes altered fear memory in male *Cntnap4* knockout mice

**DOI:** 10.1016/j.ebiom.2022.104323

**Published:** 2022-11-15

**Authors:** Wenlong Zhang, Jie Huang, Feng Gao, Qianglong You, Liuyan Ding, Junwei Gong, Mengran Zhang, Runfang Ma, Shaohui Zheng, Xiangdong Sun, Yunlong Zhang

**Affiliations:** a Department of Neurology, The First Affiliated Hospital of Guangzhou Medical University, Guangzhou, 510120, China; b Department of Neurology, Institute of Neuroscience, Key Laboratory of Neurogenetics and Channelopathies of Guangdong Province and the Ministry of Education of China, The Second Affiliated Hospital, Guangzhou Medical University, Guangzhou, 510260, China

**Keywords:** Autism spectrum disorder, Cntnap4, Fear memory, GABAergic transmission, Gut microbiome

## Abstract

**Background:**

Autism spectrum disorder (ASD) is a common neurodevelopmental disease, characterized by deficits in social communication, restricted and repetitive behaviours, and impaired fear memory processing. Severe gastrointestinal dysfunction and altered gut microbiome have been reported in ASD patients and animal models. Contactin associated protein-like 4 (*CNTNAP4*) has been suggested to be a novel risk gene, though its role in ASD remains unelucidated.

**Methods:**

Cntnap4^−/−^ mice were generated to explore its role in ASD-related behavioural abnormalities. Electrophysiological recording was employed to examine GABAergic transmission in the basolateral amygdala (BLA) and prefrontal cortex. RNA-sequencing was performed to assess underlying mechanisms. 16S rDNA analysis was performed to explore changes in faecal microbial composition. Male Cntnap4^−/−^ mice were fed with *Lactobacillus reuteri* (*L. reuteri*) or faecal microbiota to evaluate the effects of microbiota supplementation on the impaired fear conditioning mediated by *Cntnap4* deficiency.

**Findings:**

Male Cntnap4^−/−^ mice manifested deficiency in social behaviours and tone-cue fear conditioning. Notably, reduced GABAergic transmission and GABA receptor expression were found in the BLA but not the prefrontal cortex. In addition, gut *Lactobacillus* were less abundant in male Cntnap4^−/−^ mice, and *L. reuteri* treatment or faecal microbiota transplantation rescued abnormal tone-cued fear memory and improved local GABAergic transmission in the BLA of male Cntnap4^−/−^ mice.

**Interpretation:**

Cntnap4 shapes GABAergic transmission of amygdala and fear conditioning, and microbial intervention represents a promising therapy in ASD intervention.

**Funding:**

10.13039/501100001809National Natural Science Foundation of China, Science and Technology Planning Project of Guangzhou, 10.13039/100009659Guangzhou Medical University, and 10.13039/501100002858China Postdoctoral Science Foundation.

**Research in context**

**Evidence before this study**

Accumulating evidence points to the importance of the gut microbiome in modulating social behaviours in ASD. *CNTNAP4* was identified as a novel risk gene for ASD. We previously demonstrated that *Cntnap4* deficiency contributes to the pathogenesis of neurodegenerative diseases. However, the molecular mechanism of *Cntnap4* deficiency and the role of gut microbiome dysbiosis in ASD-related behavioural abnormalities due to *Cntnap4* deficiency remain undefined.

**Added value of this study**

Our findings delineate effects of *Cntnap4* deficiency on social behaviours and tone-cue fear conditioning. Reduced GABAergic transmission in the basolateral amygdala (BLA) and decreased abundance of gut *Lactobacillus* were observed in male Cntnap4^−/−^ mice. Remarkably, *Lactobacillus reuteri* administration or faecal microbiota transplantation rescued abnormal tone-cued fear memory and improved GABAergic transmission in the BLA of male Cntnap4^−/−^ mice. These observations underscore the mediatory roles of the microbiota in linking *CNTNAP4* genetic deficiency and impaired social behaviours in ASD.

**Implications of all the available evidence**

Our findings indicate a critical role for Cntnap4 in shaping GABAergic transmission of the amygdala and fear conditioning. Additionally, as an emerging therapeutic tool to improve the behavioural abnormalities in ASD, microbial intervention represents a promising candidate therapy. Considering the growing prevalence of ASD and lack of efficient therapeutic interventions, our findings have important potential clinical applications in improving impaired social behaviours in ASD.

## Introduction

ASD is a complex neurodevelopmental disease encompassing autistic disorder, Asperger syndrome, childhood disintegrative disorder, and pervasive developmental disorder not otherwise specified, according to the Diagnostic and Statistical Manual of Mental Disorders, 5th ed. (DSM-5) definition.^1^ The key diagnostic features of ASD patients include deficits in social communication and restricted, repetitive patterns of behaviour, interest, or activities.^1^, ^2^, ^3^, ^4^ Several brain regions, such as the cerebral cortex, cerebellum, amygdala and hippocampus, underlie ASD.^5^, ^6^, ^7^ Among them, the amygdala is structurally and functionally linked with ASD, as evidenced by its reduced volume in autism patients and functional association with autism-related social behaviours.^8^, ^9^, ^10^ Because the amygdala is also important in fear memory processing,^11^^,^^12^ it may regulate fear conditioning in ASD. Abnormal fear memory alterations have been investigated in genetic and drug-induced autism models.^13^, ^14^, ^15^, ^16^ However, the underlying mechanisms of fear memory alteration in ASD remain unclear.

More than 80% of ASD has high heritability,^17^ and several risk genes, such as *NLGN3*, *MECP2*, *SHANK2*, *SHANK3*, *ARID1B*, *CHD8*, and *ASH1L*, have been demonstrated to be genetically linked with ASD.^18^, ^19^, ^20^, ^21^, ^22^, ^23^, ^24^, ^25^ Among these, contactin-associated protein-like 2 (*CNTNAP2*) and *CNTNAP4* are susceptible genes, and mutation or deletion of these genes lead to autism-like phenotypes.^26^, ^27^, ^28^ As a transmembrane protein member of the neurexin superfamily, Cntnap4 is involved in neuron–glia interaction and is critical for neurological development and synaptic function.^29^^,^^30^ Utilizing its postsynaptic density-95, disks-large, and zonula occludens-1 (PDZ)-containing domain, Cntnap4 interacts with Mint1, calcium/calmodulin-dependent serine protein kinase (CASK), and ligand of Numb-protein X2 (LNX2) to regulate GABAergic (γ-aminobutyric acid producing) transmission and neuronal differentiation.^31^, ^32^, ^33^ In the brain, Cntnap4 is mainly expressed in interneurons in the olfactory bulb, hippocampus and amygdala, and dopaminergic neurons in the substantia nigra.^30^ Absence of *Cntnap4* has been observed to induce autism-related behavioural abnormality such as over-grooming by disturbing GABAergic signalling.^28^ Recently, rare copy number variations (CNV) of *CNTNAP4* and noncoding variants within promotor of *CNTNAP4* have been reported to be associated with ASD.^34^^,^^35^ Furthermore, we and other groups demonstrated that deletion of *Cntnap4* or CNV polymorphisms of *CNTNAP4* cause neurodegenerative diseases, such as Parkinson's disease (PD) and Alzheimer's disease (AD), and neurological disorders, such as epilepsy.^36^, ^37^, ^38^ Because GABAergic signalling in the amygdala is closely associated with the consolidation and extinction of fear memory,^39^ we speculated that Cntnap4 may also play a role in fear memory processing.

In addition to genetic associations, gene-environment interactions also contribute significantly to ASD pathogenesis.^40^^,^^41^ Notably, the gut microbiota is a critical player in gene–environment interactions for various human diseases.^42^^,^^43^ Previously, altered gut microorganisms and serious gastrointestinal problems have been reported in ASD patients,^44^^,^^45^ and transplantation of gut microbiota from human ASD patients has been shown to mediate autism-like abnormal behaviour and neuronal activity in germ-free mice.^46^ Furthermore, microbiota transfer therapy shows benefits in treating ASD.^47^ Intriguingly, lessons from *Cntnap2* knockout mice show segregated contributions of host genetics and the microbiome to autism-related social behaviour.^48^ Nevertheless, the role of the gut microbiota in fear memory processing in ASD has not been fully elucidated.

In the present study, we investigated the contribution of the gut microbiota in altered fear memory processing in Cntnap4^−/−^ mice. We report for that male Cntnap4^−/−^ mice manifest deficiency in social behaviours and tone-cue fear conditioning. In addition, we found reduced GABAergic transmission in the basolateral amygdala (BLA) and decreased abundance of gut *Lactobacillus* in male *Cntnap4* deficiency mice. Supplementation of *Lactobacillus reuteri* (*L. reuteri*) or faecal microbiota transplantation (FMT) restored tone-cued fear memory and GABAergic plasticity in the amygdala of male Cntnap4^−/−^ mice. Together, these findings suggest that Cntnap4 shapes GABAergic transmission in the amygdala and fear conditioning, for which the gut microbiota provides a promising candidate for ASD intervention.

## Methods

### *L. reuteri* culture and treatment

*L. reuteri* (ATCC-PTA-6475, Guangdong Microbial Culture Collection Center, Guangzhou, China) was cultured anaerobically in de Man, Rogosa, and Sharpe (MRS) broth at 37 °C as previously described.^48^ Briefly, cultures were centrifuged, washed, resuspended in PBS, and frozen at −80 °C until use. Mice were intragastrically administered live *L. reuteri* (1 × 10^8^ organisms/mouse/day) for 4 weeks.

### Faecal microbiota transplantation

Fresh faeces were collected and transplanted to male Cntnap4^+/+^ and Cntnap4^−/−^ mice according to a previous study.^49^ Briefly, fresh stools were collected from healthy male wild-type C57BL/6J mice, and 100 mg of faeces were immediately placed into 1 mL of sterile 0.01 M PBS and steeped for 1 min. Then, the dissolved faeces were centrifuged at 900 g at 4 °C for 3 min. The suspension was collected, and bacterial suspension was then delivered to each recipient mouse (10 mL/kg) via oral gavage for seven consecutive days.

### Animals

Cntnap4^−/−^ mice were generated by Shanghai Model Organisms Center, Inc (Shanghai, China) according to our previous report.^36^ First, the mMESSAGE mMACHINE T7 Ultra Kit (Ambion, TX, USA) was used to transcribe Cas9 mRNA, and the MEGAshortscript Kit (ThermoFisher, Waltham, MA, USA) was used to transcribe four single guide RNAs (sgRNAs) targeted to delete exon 3 of *Cntnap4 gene* (sgRNA1: TGCCACTTGTGTTCATTTA GAGG; sgRNA2: TGCCTCTAAAT GAA CACAA GTGG; sgRNA3: ATGGTTTAGT GGACTCGTGTGGG; sgRNA4: CATGGTTTAGTGGACTCGTGTGG) *in vitro*. Then the Cas9 mRNA and sgRNAs were injected into zygotes of C57BL/6J mice and transferred to pseudopregnant recipients. The Cntnap4^−/−^ mice were validated by PCR and sequencing using primer pairs: F-5′-CCAAACCCAATTCATTCCTT-3′; R-5′-GCAACACTGTAAATCACG CATA-3′. Male and female Cntnap4^−/−^ mice (nearly 12–14 weeks) were double-blindly and randomly used in this study, and age-and sex-matched Cntnap4^+/+^ littermates were set as the control. Four mice were kept in each cage under a controlled 12/12-h light/dark cycle, temperature (22 ± 1 °C), relative humidity (60 ± 5%), and food and water were provided *ad libitum*. The total number of animals we used was 171 mice; there were 125 male mice (62 Cntnap4^+/+^ mice and 63 Cntnap4^−/−^ mice) and 46 female mice (25 Cntnap4^+/+^ mice and 21 Cntnap4^−/−^ mice). We calculate the samples size based on experience with the respective tests, variability of the assays, and interindividual differences within groups.

### Behavioural tests

#### Open field test (OFT)

The OFT was performed according to our previous study.^50^ Before the OFT, mice were habituated in the testing room for 1 day. The open field consisted of a box (40 cm × 40 cm × 40 cm) including peripheral and central sectors. Each mouse was placed individually in the central sector, and its locomotion was recorded with the EthoVision XT tracking system (Beijing, China). Behavioural parameters recorded during the 15 min test period included the total distance travelled and the time spent in the central and the peripheral zone. After each trial, the apparatus was cleaned with 75% ethanol.

#### Social interaction test (SIT)

The SIT was performed as previously described.^51^ First, the mice were placed in an empty Plexiglas arena consisting of corner zones and an interaction zone for 150 s. The interaction zone of the test arena encompasses a 14 cm × 24 cm rectangular area projecting 8 cm around the wire-mesh enclosure. The corner zones encompass a 9 cm × 9 cm area projecting from two corner joints opposing the wire-mesh enclosure. Next, a CD-1 mouse was placed within the wire-mesh enclosure base and the test mouse was placed back in the test arena for 150 s. The total distance travelled and time spent in the corner or interacting zone with the CD-1 mouse were recorded via EthoVision XT (Beijing, China).

#### Social partition test

The test was performed as previously described, with a few modifications.^52^ Each test mouse was removed from the homecage and housed individually in a new cage, which was equally separated by a perforated transparent partition (0.6 cm-diameter holes) for 3 days before the test. On the test day 1, each test mouse was housed overnight with an age- and gender-matched C57BL/6J mouse (familiar partner) placed on the other side of the partition. On the test day 2, the first 5 min activity at the partition board with the familiar partner was recorded; then the familiar partner was replaced by a novel age- and gender-matched C57BL/6J mouse (novel partner), and a 5-min interest at the partition was recorded; the novel partner was substituted by the original familiar partner, and the last 5 min activity at the partition was recorded. The time spent at the partition in each 5 min trial for the test mouse was assayed using Observer XT (EthoVision XT, Beijing, China) by a double-blind experimenter.

#### Three-chamber test

A three-chambered apparatus was used to assess the social behaviours as previously described, with a few modifications.^53^ After a 10 min habituation period in the middle chamber, each test mouse was allowed to explore all 3 empty chambers for 10 min (Phase 1). Then an age- and gender-matched C57BL/6J mouse (unfamiliar stranger 1) was placed into a wire cage, and an identical empty wire cage was placed in the opposite chamber. The test mouse was then immediately placed in the middle chamber with the 2 doorways open, a 10 min sociability test was performed and the mouse was allowed to freely explore all chambers (Phase 2). In the last session, the empty wire cage was substituted with one that another age- and gender-matched C57BL/6J mouse (unfamiliar stranger 2) was placed in, and, as described in Phase 2, a 10 min social novelty preference test was recorded (Phase 3). The time spent in each chamber was calculated and analysed using Observer XT (EthoVision XT, Beijing, China) by a double-blind experimenter.

#### Fear conditioning test

A fear conditioning test was performed according to our previous work.^12^ The fear conditioning test was performed using the NIR Video Fear Conditioning Package for Mice (Med Associates, Vermont, USA). On day 1, after a 180 s habituation, the mice were exposed to a tone (75 dB, 2800 Hz, 30 s) and then to the same tone combined with electrical shock (1 mA) for 1 s, which was repeated four times at an interval of 110 s. On day 2, the mice were placed in the same chamber as on day 1 for 5 min without tone or electrical shock to assess context-dependent fear conditioning. On day 3, the mice were placed in a black-coloured chamber of the same size to assess the baseline activity for 3 min, and then the same tone as on day 1 was applied for 3 min to assess tone-dependent fear conditioning. For evaluation of context- and tone-dependent conditioning, the freezing scores were obtained by the Video Freeze® Software system (Med Associates, Vermont, USA) and expressed as percent of the baseline activity.

#### Y maze test

A Y maze test was performed as we described previously.^54^ The Y maze consisted of three arms (30 cm long, 10 cm wide, and 20 cm high) and a connected central area. Mice were placed within the centre zoom and were allowed to explore in the Y maze freely for 8 min. A video tracking system (EthoVision XT; Beijing, China) was used to analyse spontaneous alterations.

#### Elevated plus maze (EPM)

The EPM was performed according to our previous study.^55^ The EPM device consists of two open arms (30 × 5 cm), two closed arms (30 × 5 × 15 cm), and a central zone (5 × 5 cm). The device was elevated to a height of 50 cm above the ground. Mice were placed in the central intersection for 5 min. A video tracking system (EthoVision XT) was used to record the time spent in the open and closed arms.

#### Tail suspension test (TST)

The TST was performed as we previously described.^56^ Mice were suspended 5 cm above the ground using an adhesive tape placed approximately 1 cm from the tip of the tail. The immobility time was recorded via EthoVision XT for 6 min.

### RNA-sequencing (RNA-seq)

RNA-seq was performed according to our recent work.^50^ Total RNA from the amygdala of male and female Cntnap4^+/+^ and Cntnap4^−/−^ mice was collected using Trizol (Invitrogen, Carlsbad, CA, USA). Library preparation for RNA-seq was performed using the TruSeq RNA Sample Preparation Kit (Illumina, San Diego, CA) with 300 ng of total RNA. Single read (45 bp) sequencing was conducted using the HiSeq 2500 platform (Illumina, San Diego, CA). Reads were aligned with STAR (v2.5.3a) against the murine reference genome by Novogene (Beijing, China). Transcripts were analysed with the Partek E/M algorithm and processed for normalization with the DEseq2 algorithm. Differentially expressed genes (DEGs) were set on batch effect (fold-change |1.5|, FDR-adjusted *p* value < 0.05). Samples were subjected to differential expression analysis with DESeq259 (v.1.14.1). Kyoto Encyclopedia of Genes and Genomes (KEGG) analysis of differentially expressed genes (DEGs) and gene ontology (GO) term enrichment analysis were performed using the R package (v 3.5.1). RNA-seq data used in this study are available under GEO: GSE208542 for male Cntnap4^+/+^ and Cntnap4^−/−^ mice and GSE208397 for female Cntnap4^+/+^ and Cntnap4^−/−^ mice.

### Quantitative real-time (RT)-PCR

Total RNA was extracted using Trizol (Invitrogen, Carlsbad, CA, USA). Afterward, RNA was transcriptionally reversed to cDNA using a Reverse Transcription Kit (QIAGEN, Waltham, MA, USA), and relative gene expressions were calculated using SYBR Green PCR Mix (Takara). Results were obtained by using the 2^−ΔΔCT^ method as we described previously.^50^ Primers used in this study are listed in Supplementary Table S1. Primers for *L. reuteri* and general bacteria were reported by Sgritta et al.^57^ GAPDH mRNA was used to normalize the levels of GABA receptors and oestrogen receptors in the amygdala, while general bacteria mRNA was used to normalize the level of *L. reuteri* in the colon.

### 16S rDNA analysis of faecal samples

Total genome DNA from faecal samples was extracted using the CTAB/SDS method.^54^ The DNA concentration and purity were examined by 1% agarose gels, and the DNA was diluted to 1 ng/μL using sterile water. 16S/18S rRNA genes were amplified used specific primers (16S V4: 515F-806R; 18S V4: 528F-706R; 18S V9: 1380F-1510R; ITS1: ITS5-1737F, ITS2-2043R; ITS2: ITS3-2024F, ITS4-2409R) with barcodes. All PCR reactions were carried out in 30 μL reactions with 15 μL of High-Fidelity PCR Master Mix (New England Biolabs [NEB], Ipswich, MA, USA), 0.2 μM forward and reverse primers, and 10 ng template DNA. Thermal cycling consisted of initial denaturation at 98 °C for 1 min, followed by 30 cycles of denaturation at 98 °C for 10 s, annealing at 50 °C for 30 s, elongation at 72 °C for 30 s, and a final elongation step at 72 °C for 5 min. Samples with bright main strips between 400 and 450 bp were chosen for further PCR quantification and qualification. Sequencing libraries were generated using the Ultra™ DNA Library Prep Kit for Illumina (NEB, Ipswich, MA, USA) according to the manufacturer's recommendations. The library quality was assessed on the Qubit2.0 Fluorometer (ThermoFisher, Waltham, MA, USA) and Agilent Bioanalyzer 2100 system (Agilent Technologies, CA, USA). Finally, the library was sequenced on an Illumina HiSeq platform (Illumina, San Diego, CA), and 250 bp paired-end reads were generated.

### Microbial analysis

Paired-end reads from the original DNA fragments were merged by using FLASH.^58^ Sequences were analysed using the Quantitative Insights Into Microbial Ecology (QIIME) software package,^59^ and in-house Perl scripts were used to analyse α- (within samples) and β- (among samples) diversity. Sequences with ≥97% similarity were assigned to the same operational taxonomic units (OTUs).^60^ QIIME calculates both weighted and unweighted unifrac for Principal Coordinate Analysis (PCoA) and Unweighted Pair Group Method with Arithmetic mean (UPGMA) clustering. To further explore the data of microbial diversity of the differences among the samples, significance tests were conducted with statistical analysis methods, including T-test, MetaStat, LEfSe, Anosim and MRPP.

### Whole-cell electrophysiological recording

Electrophysiological recording was performed as previously reported.^55^^,^^61^ Briefly, mice were anesthetized with isoflurane. Brains were rapidly removed and chilled in ice-cold modified artificial cerebrospinal fluid (ACSF) containing (in mM): 120 Choline-Cl, 2.5 KCl, 7 MgCl_2_, 0.5 CaCl_2_, 1.25 NaH_2_PO_4_, 25 NaHCO_3_, and 10 Glucose. Coronal BLA and prefrontal cortical (PrL) slices of 300 μm thickness were generated in ice-cold modified ACSF using a vibratome (Leica VT-1000S, Germany) and transferred to normal ACSF containing (in mM): 126 NaCl, 3 KCl, 1 MgSO_4_, 2 CaCl_2_, 1.25 NaH_2_PO_4_, 26 NaHCO_3_, and 10 Glucose. The brain slices were incubated at 32 °C for 30 min and at room temperature for an additional 1 h before recording. All liquids were saturated with 95% O_2_/5% CO_2_ (vol/vol).

Brain slices were then transferred to a recording chamber, and the ACSF was continuously overlaid at a flow rate of 2 mL/min. To record the spontaneous inhibitory postsynaptic current (sIPSC), PrL 2–3 layer and BLA pyramidal neurons were held at −70 mV in the presence of 20 μM CNQX and 100 μM AP-5. The pipette solution contained (in mM): 140 CsCl, 10 Hepes, 0.2 EGTA, 1 MgCl_2_, 4 Mg-ATP, 0.3 Na-GTP, 10 phosphocreatine and 5 QX314 (pH 7.4, 285 mOsm). Miniature events were analysed with the MiniAnalysis program (Synaptosoft Inc., NJ, USA). For spontaneous excitatory postsynaptic current (sEPSC) recording, the PrL 2–3 layer and BLA pyramidal neurons were held at −70 mV in the presence of 20 μM RS-95531. The pipette solution contained (in mM): 125 Cs-methanesulfonate, 5 CsCl, 10 Hepes, 0.2 EGTA, 1 MgCl_2_, 4 Mg-ATP, 0.3 Na-GTP, 10 phosphocreatine and 5 QX314 (pH 7.4, 285 mOsm).

In all experiments, the series resistance was maintained below 20 MΩ and not compensated. The cells were eliminated if series resistance fluctuated more than 20% of initial values. Data were acquired with a MultiClamp 700B amplifier and 1440A digitizer (Molecular Devices, San Jose, CA, USA), which were filtered at 1 kHz and sampled at 10 kHz.

### Immunofluorescence

Mouse brains were fixed in 4% paraformaldehyde and sectioned with a freezing microtome (Leica, Hamburg, Germany). The sections were blocked with 5% bovine serum albumin and then incubated overnight at 4 °C with the primary antibodies, c-Fos (Cat# sc-271243; Research Resource Identifiers [RRID]: AB_10610067, Santa Cruz Biotechnology, Dallas, TX, USA), and parvalbumin (PV, #235; RRID not available, Swant Inc., Rte Ancienne Papeterie Marly Innovation Center, Marly, Switzerland). The appropriate species of Alexa Fluor 488/594-conjugated secondary antibodies, goat anti-mouse IgG (H + L) (Cat# 70-GAM4882; RRID not available, Multi Sciences, Hangzhou, China) and goat anti-rabbit IgG (H + L) (Cat# 70-GAR5942; RRID not available, Multi Sciences, Hangzhou, China), were then applied to the sections. Images were obtained on a confocal Leica microscope (SP8; Leica, Hamburg, Germany). Image analysis was performed by ImageJ software (National Institutes of Health, Bethesda, MD, USA).

### Western blotting

The amygdala tissues were collected, and the proteins were extracted using RIPA Lysis Buffer containing protease inhibitors (Beyotime Biotechnology, Shanghai, China). The protein samples were separated on 4–20% Tris-glycine and transferred to polyvinylidene difluoride membranes. The membranes were blocked with 5% bovine serum albumin and incubated with different primary antibodies, Cntnap4 (Cat# orb544737; RRID not available, Biorbyt LLC, San Francisco, CA, USA), GABA_A_Rα1 (Cat# 06-868; RRID: AB_310272, Millipore, Billerica, MA, USA), GABA_A_Rα2 (Cat# ab193311; RRID: AB_2890213, Abcam, Cambridge, MA, USA), GABA_A_α5 (Cat# sc-393921; RRID not available, Santa Cruz Biotechnology, Dallas, TX, USA), GABA_A_Rβ3 (Cat# sc-376252; RRID: AB_11012142, Santa Cruz Biotechnology, Dallas, TX, USA), GABA_B_R1 (Cat# sc-166408; RRID: AB_2108175, Santa Cruz Biotechnology, Dallas, TX, USA), GAPDH (Cat# 60004-1; RRID: AB_2107436, Proteintech Group, Rosemont, IL, USA). After incubation with horseradish peroxidase (HRP)-labelled secondary antibodies, goat anti-rabbit IgG (Cat# A0208; RRID: AB_2892644, Beyotime Biotechnology, Shanghai, China) and goat anti-mouse IgG (Cat# A0216; RRID: AB_2860575, Beyotime Biotechnology, Shanghai, China), images were taken on the GeneGnome XRQ Chemiluminescence imaging system (Gene Company, Hong Kong, China). Protein levels were normalized against GAPDH. Quantification was performed using ImageJ software (National Institutes of Health, Bethesda, MD, USA).

### Statistical analysis

Data are presented as mean ± standard error of the mean (SEM). In this study, we performed the Shapiro–Wilk test to evaluate the normality of parameters. The parameters with *p* > 0.05 were considered according to the normal distribution and reaching the condition of parametric methods. Sample sizes were chosen based on the means and variation of preliminary data to achieve at least 80% power and allow for a 5% type I error. All calculations for sample sizes were done using an online sample size calculator (https://clincalc.com/stats/samplesize.aspx). Data were analysed using one-way and two-way ANOVA followed by the Tukey's multiple comparisons test or Student's *t*-test, as appropriate. Differences with a *p*-value *<* 0.05 were considered statistically significant. Statistical analyses were performed using GraphPad Prism 9.0 (GraphPad Software, La Jolla, CA, USA). *P*-values are represented as ∗*p <* 0.05 and ∗∗*p <* 0.01.

### Ethics statement

All animal experimental procedures were performed in accordance with the protocols approved by the Institutional Animal Care and Use Committee of Guangzhou Medical University (Approval number: GY2020-041) and National Institute of Health guidelines on the care and use of animals (NIH Publications No. 8023, revised 1978).

### Role of funders

The funders had no role in study design, data collection, data analysis, interpretation, writing of the manuscript, or decision to submit the paper for publication.

## Results

### Male *Cntnap4* deficient mice show impaired social behaviour and tone-cued fear conditioning

**Fig. 1:**
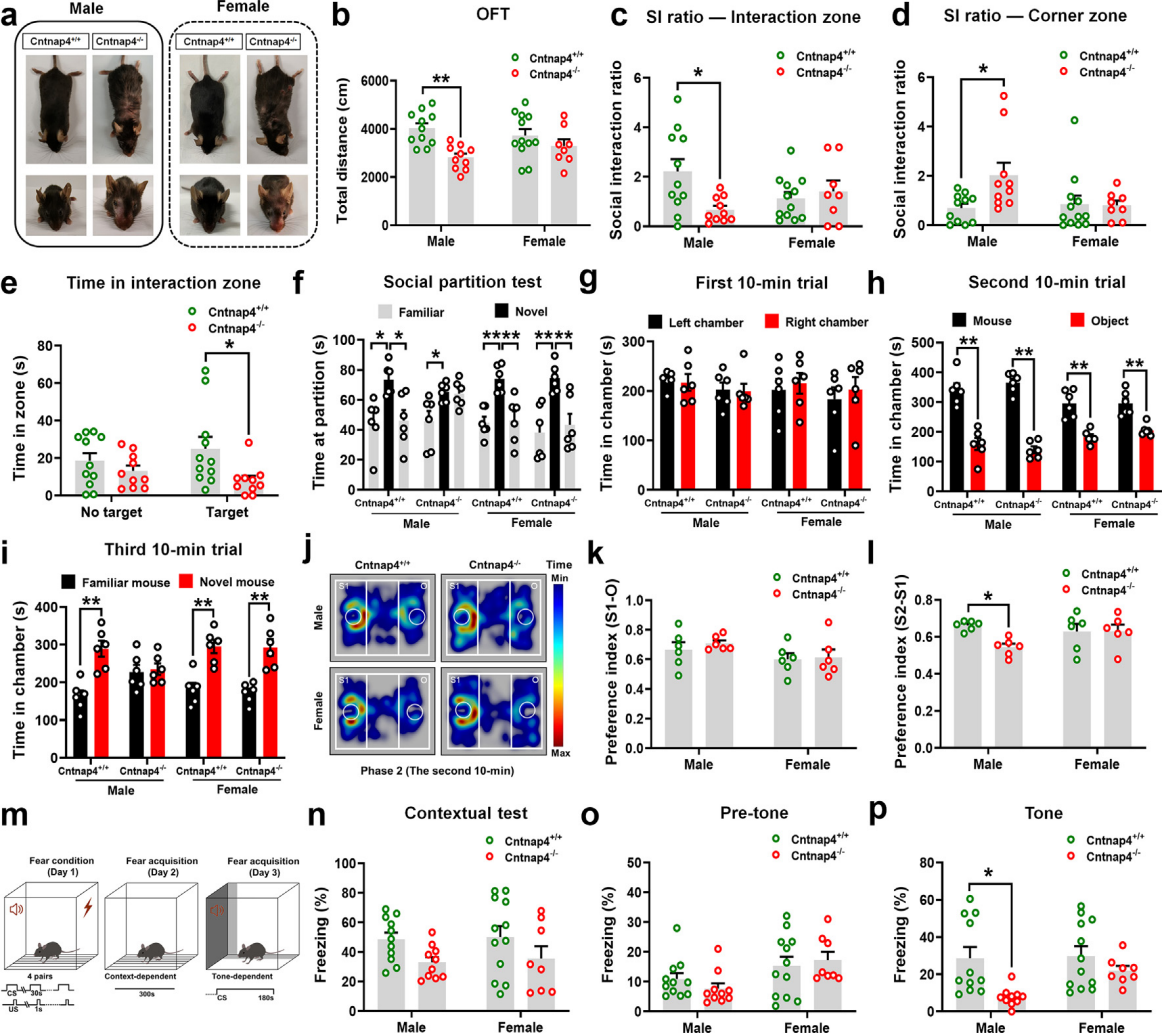
**Male Cntnap4^−/−^ mice display deficits in social behaviours and tone-cued fear conditioning.** (a) Face and body hair loss and lesions due to over-grooming in Cntnap4^−/−^ mice. (b) Total travelled distance in the open field test (OFT). *F*_1, 37_ = 2.716, *p* = 0.1078 for sex–genotype interaction; *F*_1, 37_ = 0.1366, *p* = 0.7138 for genotype; *F*_1, 37_ = 12.14, *p* = 0.0013 for sex. (c and d) Social interaction (SI) ratio in the interaction and corner zone for male and female Cntnap4^+/+^ and Cntnap4^−/−^ mice. (c) *F*_1, 37_ = 6.263, *p* = 0.0169 for sex–genotype interaction; *F*_1, 37_ = 0.2187, *p* = 0.6428 for genotype; *F*_1, 37_ = 3.063, *p* = 0.0884 for sex. (d) *F*_1, 37_ = 4.000, *p* = 0.0529 for sex–genotype interaction; *F*_1, 37_ = 2.426, *p* = 0.1279 for genotype; *F*_1, 37_ = 3.463, *p* = 0.0707 for sex. (e) Time spent in the interaction zone for male Cntnap4^+/+^ and Cntnap4^−/−^ mice with or without a target. *F*_1, 37_ = 1.728, *p* = 0.1966 for sex–genotype interaction; *F*_1, 37_ = 0.02267, *p* = 0.8811 for genotype; *F*_1, 37_ = 6.619, *p* = 0.0141 for sex. (f) Social partition test of male and female Cntnap4^+/+^ and Cntnap4^−/−^ mice facing familiar and novel mice. Male Cntnap4^+/+^ mice, *F*_2, 15_ = 6.763, *p* = 0.0081; male Cntnap4^−/−^ mice, *F*_2, 15_ = 5.443, *p* = 0.0167; female Cntnap4^+/+^ mice, *F*_2, 15_ = 12.08, *p* = 0.0007; female Cntnap4^−/−^ mice, *F*_2, 15_ = 9.434, *p* = 0.0022. (g–i) In the three-chambered social approach task, time spent in chambers during different 10-min trials. (g) Male Cntnap4^+/+^ and Cntnap4^−/−^ mice: *F*_1, 20_ = 0.003266, *p* = 0.9550 for orientation–genotype interaction; *F*_1, 20_ = 1.791, *p* = 0.1958 for orientation; *F*_1, 20_ = 0.08191, *p* = 0.7777 for genotype; female Cntnap4^+/+^ and Cntnap4^−/−^ mice: *F*_1, 20_ = 0.01495, *p* = 0.9039 for orientation–genotype interaction; *F*_1, 20_ = 0.4539, *p* = 0.5082 for orientation; *F*_1, 20_ = 0.5047, *p* = 0.4856 for genotype. (h) Male Cntnap4^+/+^ and Cntnap4^−/−^ mice: *F*_1, 20_ = 1.538, *p* = 0.2293 for target–genotype interaction; *F*_1, 20_ = 0.01927, *p* = 0.8910 for target; *F*_1, 20_ = 140.6, *p* < 0.0001 for genotype; female Cntnap4^+/+^ and Cntnap4^−/−^ mice: *F*_1, 20_ = 0.6996, *p* = 0.4128 for target–genotype interaction; *F*_1, 20_ = 0.6795, *p* = 0.4195 for target; *F*_1, 20_ = 54.24, *p* < 0.0001 for genotype. (i) Male Cntnap4^+/+^ and Cntnap4^−/−^ mice: *F*_1, 20_ = 10.80, *p* = 0.0037 for target–genotype interaction; *F*_1, 20_ = 0.07541, *p* = 0.7864 for target; *F*_1, 20_ = 13.97, *p* = 0.0013 for genotype; female Cntnap4^+/+^ and Cntnap4^−/−^ mice: *F*_1, 20_ = 0.04257, *p* = 0.8386 for target–genotype interaction; *F*_1, 20_ = 0.1184, *p* = 0.7343 for target; *F*_1, 20_ = 46.70, *p* < 0.0001 for genotype. (j) Trajectory diagram during the second 10-min trial. Male Cntnap4^−/−^ mice spent a similar length of time in the two chambers (k) and showed less preference for the novel mouse over the familiar mouse than the male Cntnap4^+/+^ mice (l). (k) *F*_1, 20_ = 0.1251, *p* = 0.7273 for sex–genotype interaction; *F*_1, 20_ = 3.513, *p* = 0.0756 for genotype; *F*_1, 20_ = 0.3997, *p* = 0.5344 for sex. (l) *F*_1, 20_ = 4.260, *p* = 0.0522 for sex–genotype interaction; *F*_1, 20_ = 0.8843, *p* = 0.3583 for genotype; *F*_1, 20_ = 3.902, *p* = 0.0622 for sex. (m) Schematic model of fear conditioning test. (n and o) Freezing levels of male and female Cntnap4^+/+^ and Cntnap4^−/−^ mice in the contextual test and before the tone was applied. (n) *F*_1, 37_ = 0.00767, *p* = 0.9307 for sex–genotype interaction; *F*_1, 37_ = 0.1105, *p* = 0.7414 for genotype; *F*_1, 37_ = 5.808, *p* = 0.0210 for sex. (○) *F*_1, 37_ = 1.040, *p* = 0.3145 for sex–genotype interaction; *F*_1, 37_ = 7.847, *p* = 0.0080 for genotype; *F*_1, 37_ = 0.06895, *p* = 0.7943 for sex. (p) Reduced freezing time in male Cntnap4^−/−^ mice upon tone-cued fear conditioning. *F*_1, 37_ = 1.901, *p* = 0.1762 for sex–genotype interaction; *F*_1, 37_ = 2.687, *p* = 0.1096 for genotype; *F*_1, 37_ = 9.505, *p* = 0.0039 for sex. Results are expressed as the mean ± SEM. In b–e and m–p, *n* = 11, 10, 12, 8 for male and female Cntnap4^+/+^ and Cntnap4^−/−^ mice, respectively; in f–l, *n* = 6 per group. ∗∗*p* < 0.01, ∗*p* < 0.05. Statistical significance was determined by two-way ANOVA with Tukey's multiple comparisons test for b–e and g–p and one-way ANOVA with Tukey's multiple comparisons test for f.

**Supplementary Fig. 1:**
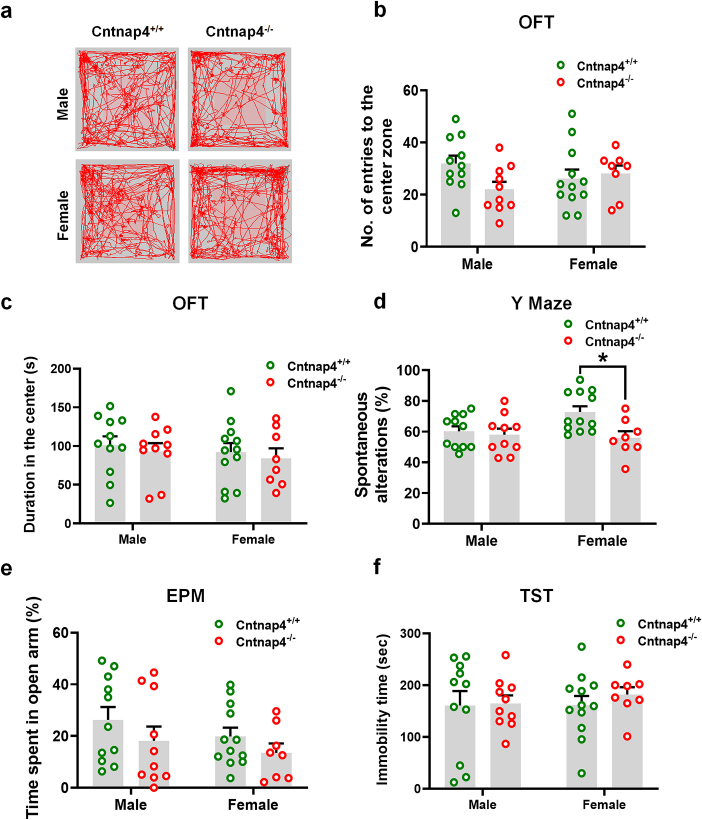


We utilized the Cntnap4^−/−^ mice previously generated by our lab^36^ to detect its mechanism in promoting autism-like behaviours. Both male and female Cntnap4^−/−^ mice displayed face and body hair loss and lesions due to over-grooming (Fig. 1a), which is consistent with previous report.^28^ Male Cntnap4^−/−^ mice travelled less distance in the open field as compared with Cntnap4^+/+^ mice (Fig. 1b and Supplementary Fig. S1a, *p* = 0.0036 for male Cntnap4^+/+^ mice vs. Cntnap4^−/−^ mice; *p* = 0.5867 for female Cntnap4^+/+^ mice vs. Cntnap4^−/−^ mice), while the number of entries and duration in the centre zone showed no obvious differences between Cntnap4^−/−^ and Cntnap4^+/+^ mice (Supplementary Fig. S1b and c). In addition, male but not female Cntnap4^−/−^ mice manifested impaired social interaction, which is a core symptom of ASD that was indicated experimentally by a reduced social interaction ratio, an increased ratio in the corner, and reduced time in the interaction zone (Fig. 1c and d, *p* = 0.0197 and 0.0394 for male Cntnap4^+/+^ mice vs. Cntnap4^−/−^ mice; *p* = 0.9536 and 0.9997 for female Cntnap4^+/+^ mice vs. Cntnap4^−/−^ mice, respectively; Fig. 1e, *p* = 0.8101 and *p* = 0.0433 for male Cntnap4^+/+^ mice vs. Cntnap4^−/−^ mice in no target and target conditions). We further performed a social partition test to assess social interaction and recognition in mice. In the social partition test, female Cntnap4^+/+^ and Cntnap4^−/−^ mice and male Cntnap4^+/+^ mice exhibited a significant increase in time spent with novel mice at the perforated partition, in contrast to the time spent with familiar mice (Fig. 1f, male Cntnap4^+/+^ mice: *p* = 0.0138 and 0.0187 for facing novel mice vs. familiar mice and original familiar mice vs. novel mice; female Cntnap4^+/+^ mice: *p* = 0.0015 and 0.0024 for facing novel mice vs. familiar mice and original familiar mice vs. novel mice; female Cntnap4^−/−^ mice: *p* = 0.0030 and 0.0096 for facing novel mice vs. familiar mice and original familiar mice vs. novel mice). While male Cntnap4^−/−^ mice did not show differential interest in familiar and novel mice, based on the time spent at the perforated partition (Fig. 1f, male Cntnap4^−/−^ mice: *p* = 0.0260 and 0.9850 for facing novel mice vs. familiar mice and original familiar mice vs. novel mice). We also utilized the three-chamber test to assess the social approach. During the first 10-min trial (phase 1), all group mice spent similar time habituating and exploring bilateral chambers, which indicated a lack of side preference in the experimental environment (Fig. 1g). Then mice were subjected to a test of sociability. During phase 2, both male and female Cntnap4^−/−^ mice did not show a significant difference from Cntnap4^+/+^ mice in the time spent with an inanimate object (Fig. 1h, *p* < 0.0001 for male Cntnap4^+/+^ and Cntnap4^−/−^ mice, *p* < 0.0001 and *p* = 0.0009 for female Cntnap4^+/+^ and Cntnap4^−/−^ mice; j and k, *p* = 0.8970 and 0.9972 for male and female Cntnap4^+/+^ mice vs. Cntnap4^−/−^ mice). During phase 3, once a novel social partner was placed in the empty wire cage, the male Cntnap4^−/−^ mice still spent a similar length of time in the two chambers (Fig. 1i, *p* = 0.0004 and *p* = 0.9885 for male Cntnap4^+/+^ and Cntnap4^−/−^ mice) and showed less preference for the novel mouse over the familiar mouse than the male Cntnap4^+/+^ mice (Fig. 1l, *p* = 0.0445 for male Cntnap4^+/+^ mice vs. Cntnap4^−/−^ mice). Furthermore, the female Cntnap4^−/−^ mice were more likely to interact with novel stranger mice compared with the Cntnap4^+/+^ mice (Fig. 1i, *p* = 0.0008 and *p* = 0.0004 for female Cntnap4^+/+^ and Cntnap4^−/−^ mice; 1l, *p* > 0.9999 for female Cntnap4^+/+^ mice vs. Cntnap4^−/−^ mice). To determine if *Cntnap4* deletion regulates the function of the amygdala, we examined tone-cued fear conditioning in Cntnap4^−/−^ mice. The mice were subjected to 4 pairs of tone and foot shock during the training day, and the freezing levels in a new environment in a probe test were examined (Fig. 1m). Both male and female Cntnap4^+/+^ and Cntnap4^−/−^ mice showed unchanged freezing levels in the contextual test and before the tone was applied (Fig. 1n and o). However, the freezing time was decreased in male Cntnap4^−/−^ mice as compared with Cntnap4^+/+^ mice in the presence of a tone, suggesting that tone-cued fear conditioning was impaired in male Cntnap4^−/−^ mice (Fig. 1p, *p* = 0.0134 for male Cntnap4^+/+^ mice vs. Cntnap4^−/−^ mice; *p* = 0.6434 for female Cntnap4^+/+^ mice vs. Cntnap4^−/−^ mice). Moreover, spontaneous alterations in the Y maze test were impaired in female Cntnap4^−/−^ mice, suggesting that the working memory may have been disturbed (Supplementary Fig. S1d, *p* = 0.9742 for male Cntnap4^+/+^ mice vs. Cntnap4^−/−^ mice; *p* = 0.0226 for female Cntnap4^+/+^ mice vs. Cntnap4^−/−^ mice). Both Cntnap4^+/+^ and Cntnap4^−/−^ mice showed no obvious changes in the elevated plus maze and tail suspension test (Supplementary Fig. S1e and f). These observations indicate that social behaviour and tone-cued fear conditioning are impaired in male *Cntnap4* deficient mice.

### Cntnap4 is critical for GABAergic transmission in the amygdala

**Fig. 2:**
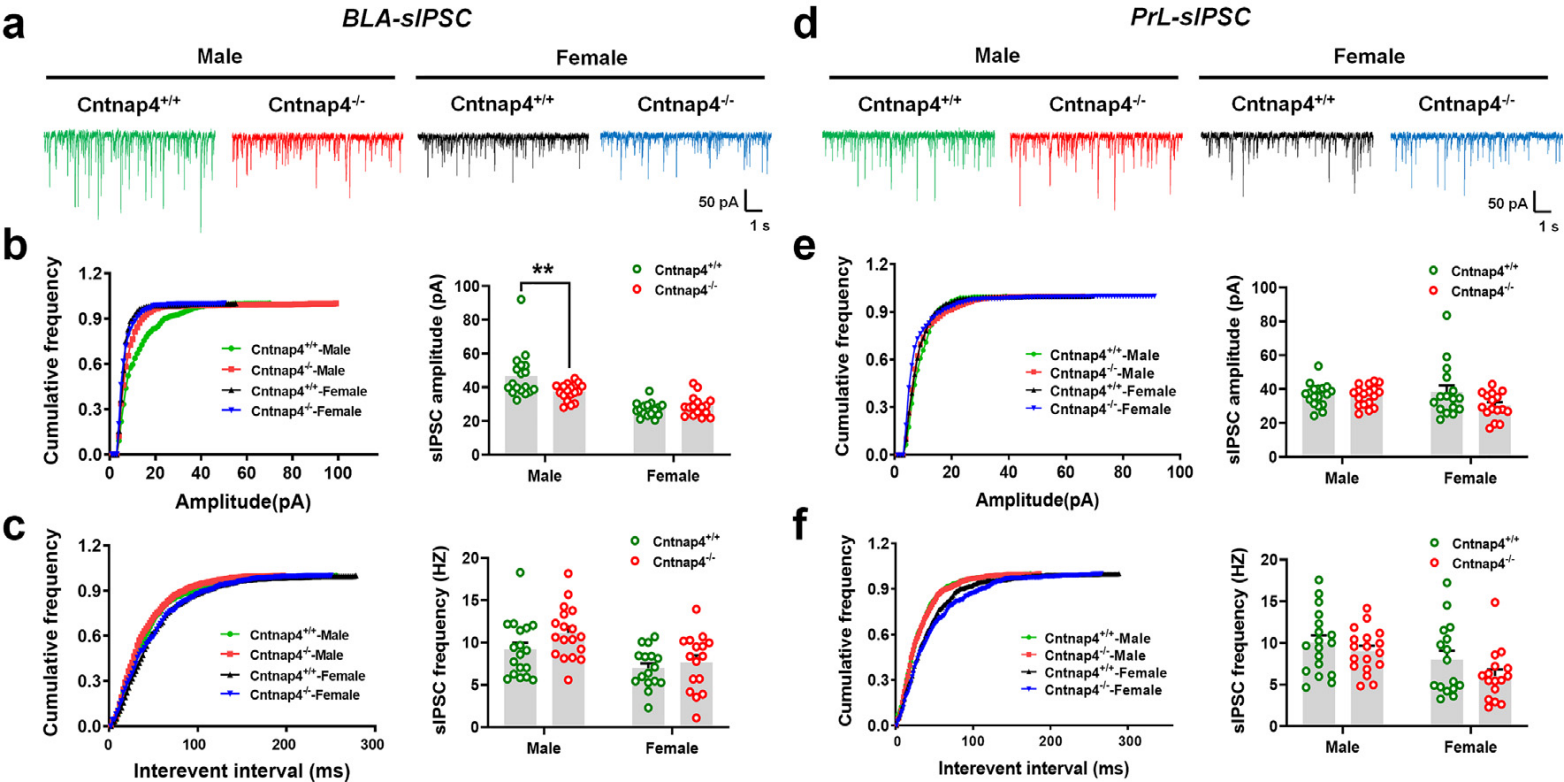
**The frequency of sIPSC in the BLA is decreased in male Cntnap4^−/−^ mice.** (a and d) Representative traces of GABA receptor-mediated sIPSCs in the BLA and PrL. All sIPSCs were recorded at a holding potential of −70 mV. (b and e) Cumulative frequency plots (left) and quantitative analysis (right) of the amplitude of GABA receptor-mediated sIPSCs in the BLA and PrL. (b) *F*_1, 64_ = 7.766, *p* = 0.0070 for sex–genotype interaction; *F*_1, 64_ = 49.67, *p* < 0.0001 for genotype; *F*_1, 64_ = 3.469, *p* = 0.0671 for sex. (e) *F*_1, 64_ = 2.716, *p* = 0.1042 for sex–genotype interaction; *F*_1, 64_ = 0.7950, *p* = 0.3759 for genotype; *F*_1, 64_ = 3.185, *p* = 0.0791 for sex. (c and f) Cumulative frequency plots of the interevent interval (left) and quantitative analysis of the frequency of GABA receptor-mediated sIPSCs (right) in the BLA and PrL. (c) *F*_1, 64_ = 0.6972, *p* = 0.4068 for sex–genotype interaction; *F*_1, 64_ = 14.81, *p* = 0.0003 for genotype; *F*_1, 64_ = 3.143, *p* = 0.0810 for sex. (f) *F*_1, 64_ = 0.3361, *p* = 0.5641 for sex–genotype interaction; *F*_1, 64_ = 8.825, *p* = 0.0042 for genotype; *F*_1, 64_ = 3.048, *p* = 0.0856 for sex. Results are expressed as the mean ± SEM. 16–18 slices from *n* = 4 mice per group. ∗∗*p* < 0.01 vs. Cntnap4^+/+^ mice. Statistical significance was determined by two-way ANOVA + Bonferroni's multiple comparisons test.

**Supplementary Fig. 2:**
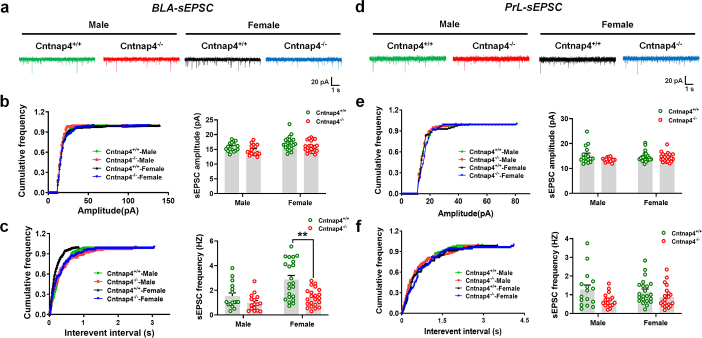


Because the BLA-prefrontal cortex (PFC) circuitry plays a distinct role in regulating fear memory processing,^62^^,^^63^ we investigated the effect of *Cntnap4* deficiency on synaptic transmission in the BLA and PFC. The sIPSC amplitude in the BLA was decreased in male Cntnap4^−/−^ compared with Cntnap4^+/+^ mice (Fig. 2a and b, *p* = 0.0065 for male Cntnap4^+/+^ mice vs. Cntnap4^−/−^ mice; *p* = 0.9203 for female Cntnap4^+/+^ mice vs. Cntnap4^−/−^ mice). On the other hand, the sIPSC frequency in the BLA and the sIPSC amplitude and frequency in the prefrontal cortical PrL showed no obvious changes in male and female Cntnap4^−/−^ mice (Fig. 2c–f). Additionally, the sEPSC frequency in the BLA was reduced in female Cntnap4^−/−^ compared with Cntnap4^+/+^ mice, while the sEPSC amplitude in the BLA and sEPSC amplitude and frequency in the PrL were unaltered in male and female Cntnap4^−/−^ mice (Supplementary Fig. S2a–f, *p* = 0.3595, 0.5052, 0.0978, and 0.0954 for male Cntnap4^+/+^ mice vs. Cntnap4^−/−^ mice; *p* = 0.2862, 0.0001, 0.9745, and 0.4567 for female Cntnap4^+/+^ mice vs Cntnap4^−/−^ mice in Fig. 2b, c, e, and f, respectively). These results suggest that *Cntnap4* deficiency may affect amygdaloid GABAergic transmission in male mice.

**Fig. 3:**
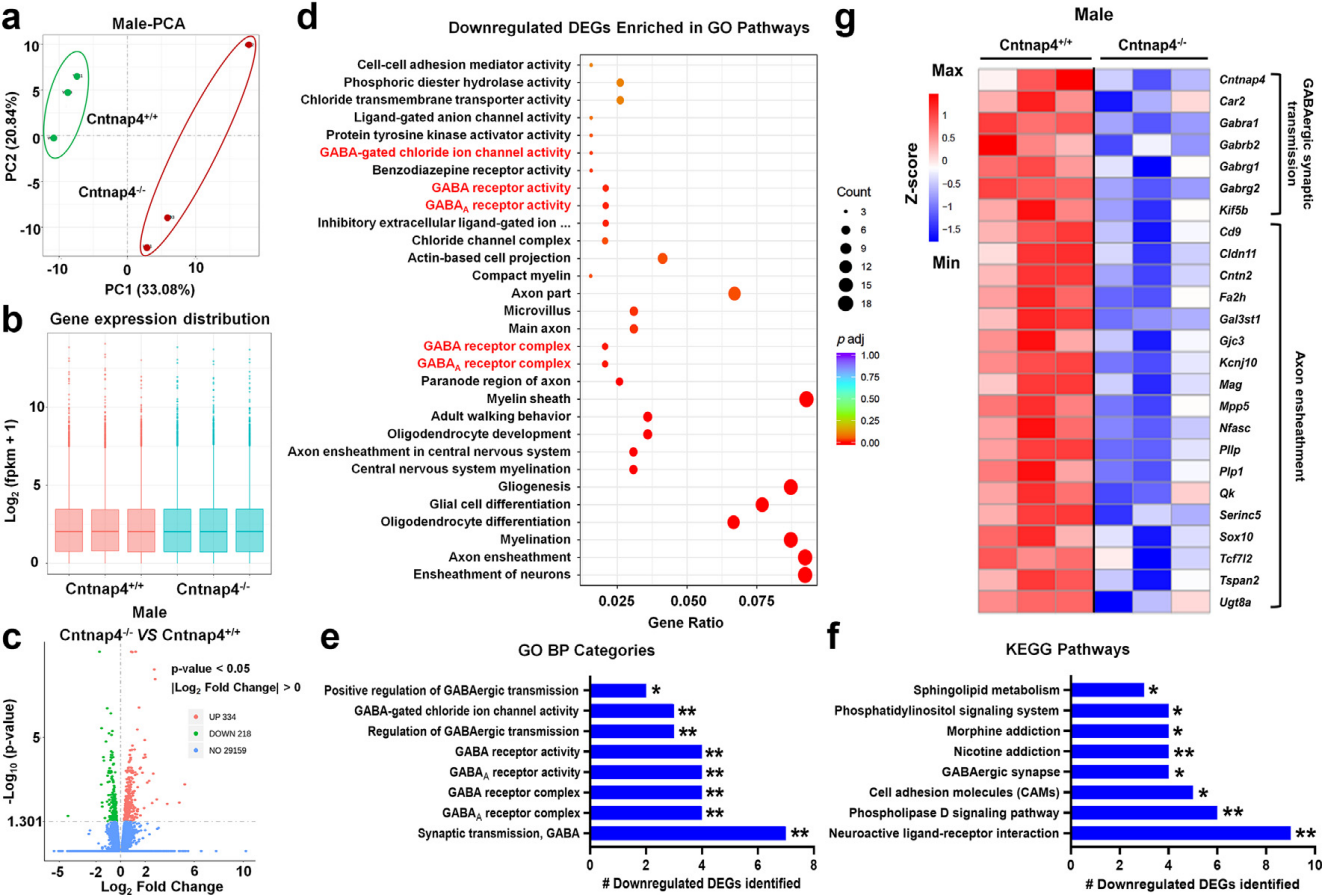
**Transcriptome analysis of amygdala DEGs in male Cntnap4^−/−^ mice.** (a) PCA score plots revealing a distinct separation of components in Cntnap4^+/+^ and Cntnap4^−/−^ mice. (b) Gene expression distribution of DEGs in Cntnap4^+/+^ and Cntnap4^−/−^ mice. (c) Volcano plot showing the DEGs between Cntnap4^+/+^ and Cntnap4^−/−^ mice. (d) GO pathways enriched by downregulated DEGs in Cntnap4^−/−^ mice compared with Cntnap4^+/+^ mice. GABAergic pathways are highlighted in red. (e and f) Representative GO and KEGG pathways enriched by downregulated DEGs. (g) Hierarchical clustering of 25 downregulated DEGs enriched in GABAergic synaptic transmission and axon ensheathment processes.

**Supplementary Fig. 3:**
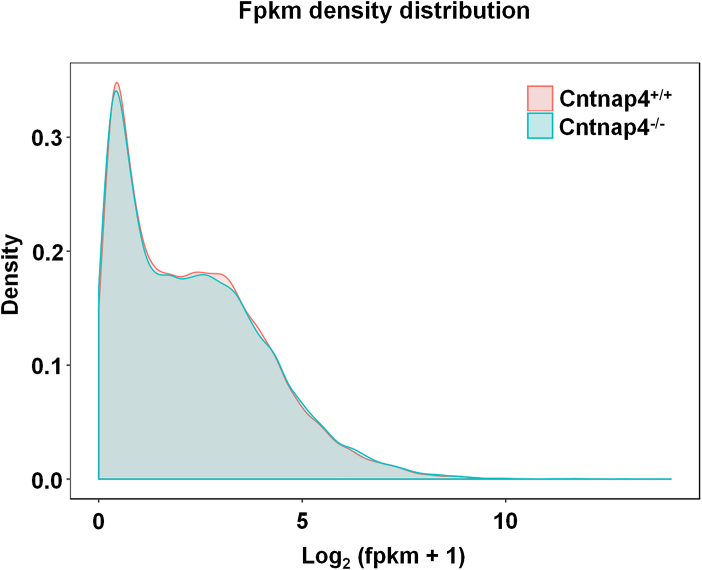


**Supplementary Fig. 4:**
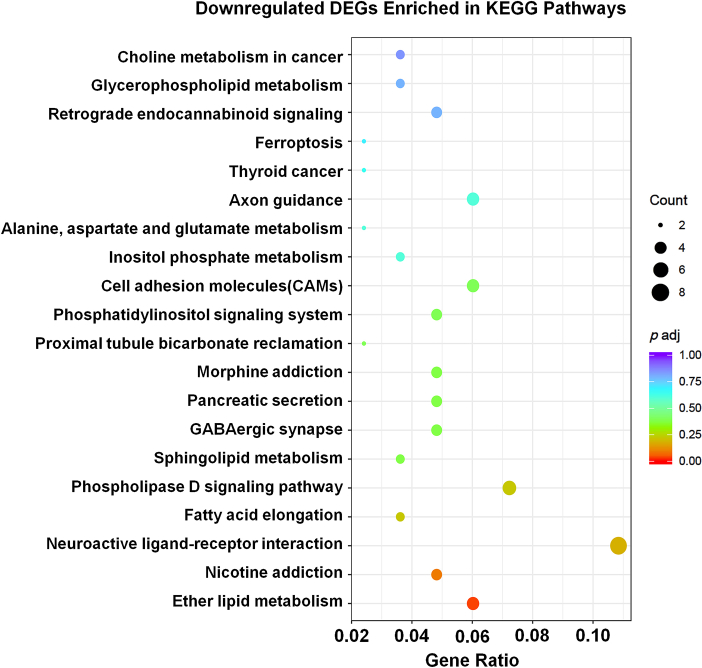


**Supplementary Fig. 5:**
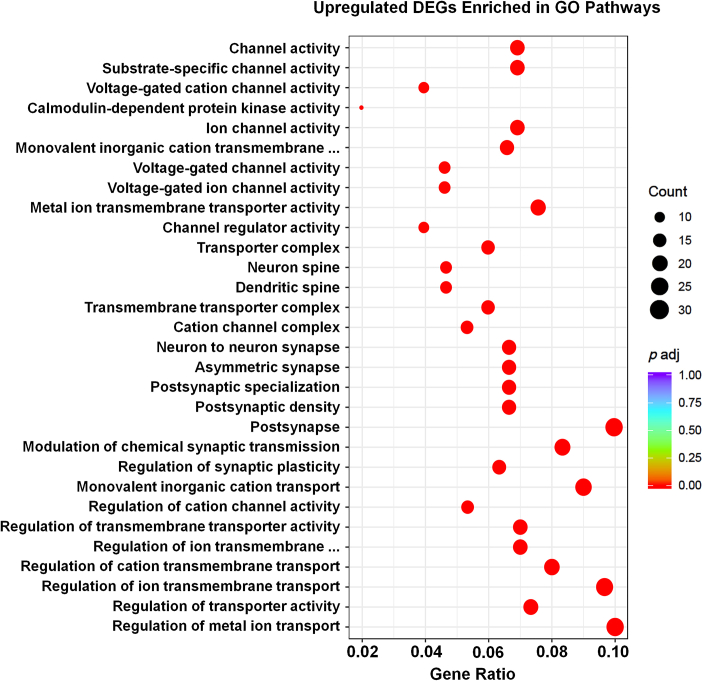


**Supplementary Fig. 6:**
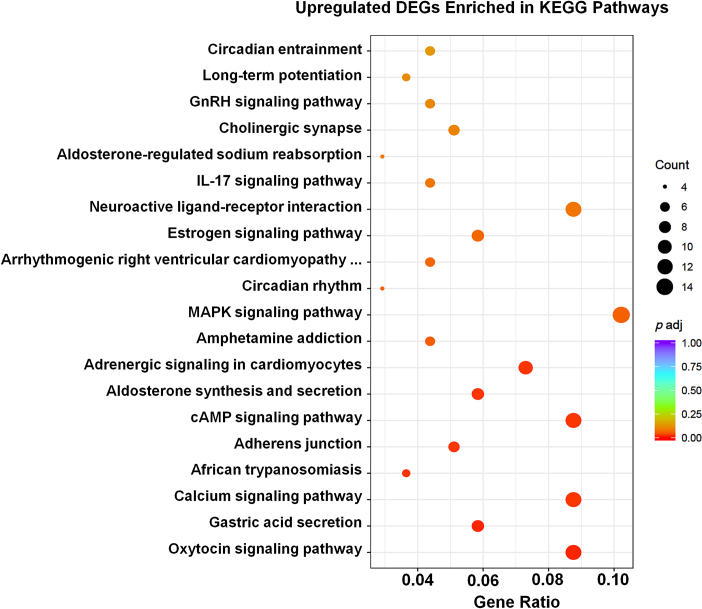


Given that male Cntnap4^−/−^ mice display an impaired fear conditioning phenotype, we further explored the effects of *Cntnap4* deficiency on amygdaloid gene expression pathways by RNA-sequencing. PCA score plots revealed a distinct separation of components in the male Cntnap4^+/+^ and Cntnap4^−/−^ groups (Fig. 3a). The gene expression distribution and FPKM density distribution appeared largely similar (Fig. 3b and Supplementary Fig. S3). However, volcano plots revealed 552 DEGs (334 increased and 218 decreased) between male Cntnap4^+/+^ and Cntnap4^−/−^ mice (Fig. 3c). The downregulated DEGs were enriched in GO and KEGG pathways related to GABAergic transmission, such as “GABAergic synapse”, “GABA receptor complex”, “GABA_A_ receptor complex”, “GABA receptor activity”, “GABA_A_ receptor activity” and “GABA-gated chloride ion channel activity” (Fig. 3d–f, Supplementary Fig. S4). Individual downregulated DEGs enriched in “GABAergic transmission and axon ensheathment” are shown in a heatmap (Fig. 3g). Specifically, *Cntnap4*, *Car2*, *Gabra1*, *Gabrb2*, *Gabrg1*, *Gabrg2* and *Kif5b* were downregulated DEGs related to GABAergic synaptic transmission in male Cntnap4^−/−^ mice. Likewise, GO and KEGG pathways enriched by upregulated DEGs are consistent with the electrophysiological recording data (Supplementary Figs. S5 and S6), thus further supporting the role for Cntnap4 in GABAergic transmission.

**Fig. 4:**
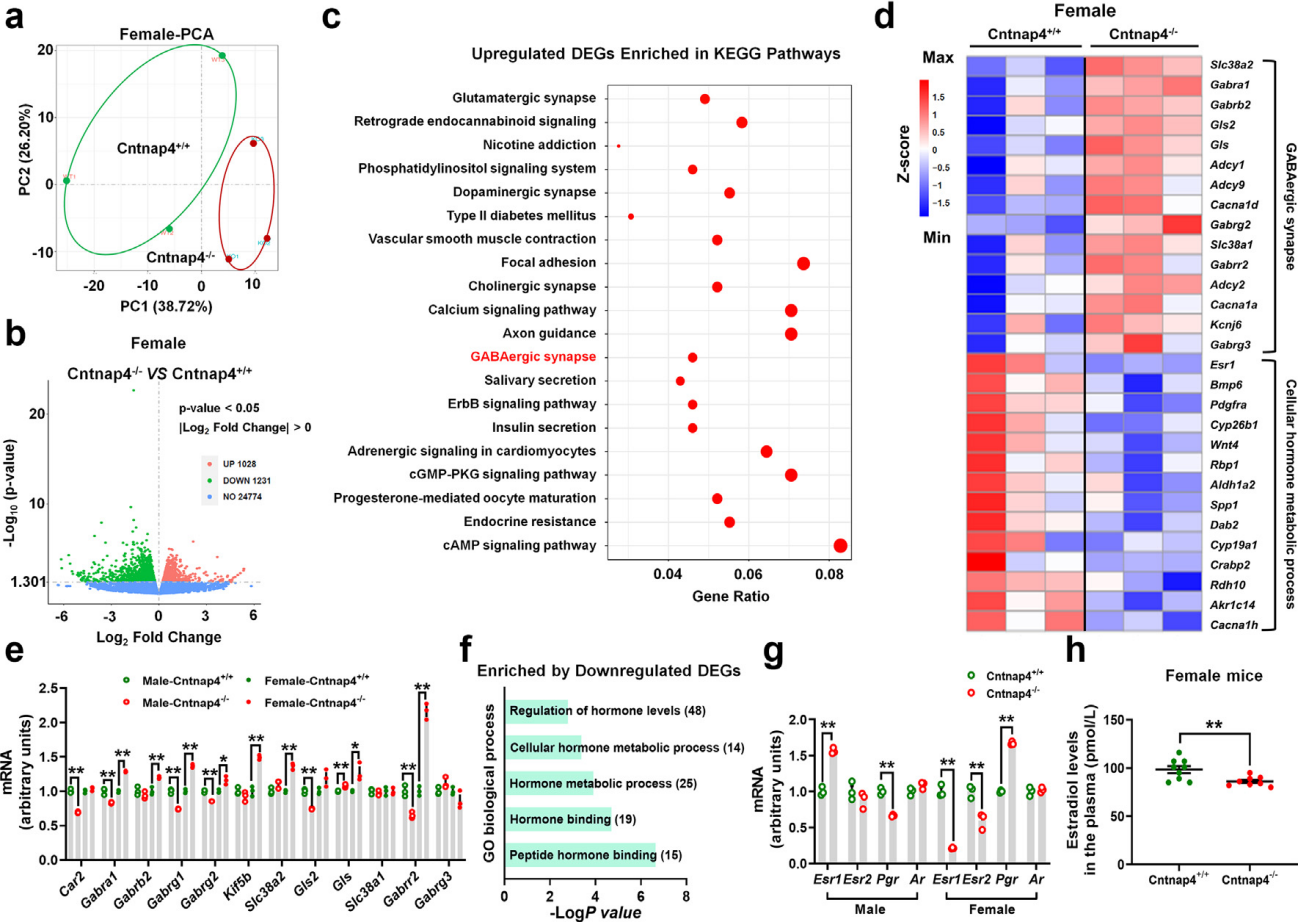
**Transcriptome analysis of amygdala DEGs in female Cntnap4^−/−^ mice.** (a) PCA score plots revealing a distinct separation of components in Cntnap4^+/+^ and Cntnap4^−/−^ mice. (b) Volcano plot showing the DEGs between Cntnap4^+/+^ and Cntnap4^−/−^ mice. (c) KEGG pathways enriched by upregulated DEGs in Cntnap4^−/−^ mice compared with Cntnap4^+/+^ mice. The GABAergic synapse is highlighted in red. (d) Hierarchical clustering of 15 upregulated DEGs enriched in GABAergic synapse and 14 downregulated DEGs enriched in the cellular hormone metabolic process. (e) The mRNA expressions of *Car2*, *Gabra1*, *Gabrb2*, *Gabrg1*, *Gabrg2*, *Kif5b*, *Slc38a2*, *Gls2*, *Gls*, *Slc38a1*, *Gabrr2*, and *Gabrg3* in the amygdala of male and female Cntnap4^+/+^ and Cntnap4^−/−^ mice. *n* = 3. (f) Representative GO pathways enriched by downregulated DEGs. (g) The mRNA expressions of *Esr1*, *Esr2*, *Pgr*, and *Ar* in the amygdala of male and female Cntnap4^+/+^ and Cntnap4^−/−^ mice. *n* = 3. (h) The estradiol levels in the plasma of female Cntnap4^+/+^ and Cntnap4^−/−^ mice. *n* = 9 and 8 for Cntnap4^+/+^ and Cntnap4^−/−^ mice. Results are expressed as the mean ± SEM. ∗∗*p* < 0.01 vs. Cntnap4^+/+^ mice. Statistical significance was determined by Student's *t*-test.

**Supplementary Fig. 7:**
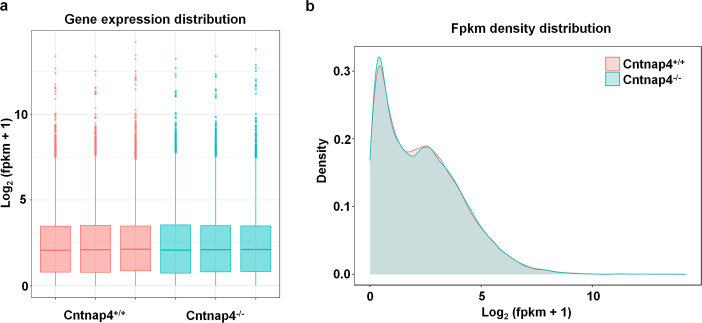


To understand the mechanism behind the sex differences in the behaviours, we also studied the transcriptomic alteration in the female Cntnap4^−/−^ mice. PCA score plots showed a distinct separation of components in the Cntnap4^+/+^ and Cntnap4^−/−^ groups (Fig. 4a), and volcano plots revealed 2259 DEGs (1028 increased and 1231 decreased) between female Cntnap4^+/+^ and Cntnap4^−/−^ mice (Fig. 4b). Like the RNA-seq data of male mice, the gene expression distribution and FPKM density distribution from female RNA-seq data also appeared largely similar (Supplementary Fig. S7a and b). However, unlike RNA-seq data from male mice, the upregulated DEGs in female Cntnap4^−/−^ mice versus Cntnap4^+/+^ mice were found to be enriched in “GABAergic synapse” in KEGG pathways (Fig. 4c). Here, we showed the upregulated DEGs enriched in “GABAergic synapse” and downregulated DEGs enriched in “Cellular hormone metabolic process” in the heatmap (Fig. 4d). To identify the effects of sex differences on GABAergic transmission we observed, we evaluated the mRNA expression of the representative up- or downregulated DEGs from male and female RNA-seq data. We found that the mRNA expressions of *Car2*, *Gabra1*, *Gabrg1*, *Gabrg2*, *Gls2*, and *Gabrr2*, which are GABA receptors or related to GABA metabolism, were decreased in the amygdala of male Cntnap4^−/−^ mice (Fig. 4e, Student's *t* test, *Car2*: *t* = 16.16, df = 4, *p* < 0.0001; *Gabra1*: *t* = 6.046, df = 4, *p* = 0.0038; *Gabrg1*: *t* = 13.00, df = 4, *p* = 0.0002; *Gabrg2*: *t* = 12.77, df = 4, *p* = 0.0002; *Gls2*: *t* = 12.84, df = 4, *p* = 0.0002; *Gabrr2*: *t* = 6.612, df = 4, *p* = 0.0027). Consistent with the RNA-seq data, *Gabra1*, *Gabrb2*, *Gabrg1*, *Gabrg2*, *Kif5b*, *Slc38a2*, *Gls*, and *Gabrr2* expressions were increased in the amygdala of female Cntnap4^−/−^ mice (Fig. 4e, Student's *t* test, *Gabra1*: *t* = 22.10, df = 4, *p* < 0.0001; *Gabrb2*: *t* = 5.779, df = 4, *p* = 0.0045; *Gabrg1*: *t* = 16.39, df = 4, *p* < 0.0001; *Gabrg2*: *t* = 4.230, df = 4, *p* = 0.0134; *Kif5b*: *t* = 10.87, df = 4, *p* = 0.0004; *Slc38a2*: t = 12.68, df = 4, *p* = 0.0002; *Gls*: *t* = 3.568, df = 4, *p* = 0.0234; *Gabrr2*: *t* = 14.89, df = 4, *p* = 0.0001). Furthermore, we found that several pathways enriched by downregulated DEGs were related to hormone metabolism (Fig. 4f). We then examined the hormone receptors, such as *Esr1* (encoding oestrogen receptor 1), *Esr2* (encoding oestrogen receptor 2), *Pgr* (encoding progesterone receptor), and *Ar* (encoding androgen receptor). Intriguingly, *Esr1* expression was increased and *Pgr* expression was decreased in the amygdala of male Cntnap4^−/−^ mice versus Cntnap4^+/+^ mice (Fig. 4g, Student's *t* test, *Esr1*: *t* = 13.46, df = 4, *p* = 0.0002; *Pgr*: *t* = 11.86, df = 4, *p* = 0.0003), while *Esr1* and *Esr2* expressions were decreased and *Pgr* expression was increased in the amygdala of female Cntnap4^−/−^ mice versus Cntnap4^+/+^ mice (Fig. 4g, Student's *t* test, *Esr1*: *t* = 16.27, df = 4, *p* < 0.0001; *Esr2*: *t* = 4.950, df = 4, *p* = 0.0078; *Pgr*: *t* = 39.66, df = 4, *p* < 0.0001). Consistently, estradiol level was decreased in the plasma of female Cntnap4^−/−^ mice versus Cntnap4^+/+^ mice (Fig. 4h, Student's *t* test, *t* = 2.959, df = 15, *p* = 0.0098).

**Fig. 5:**
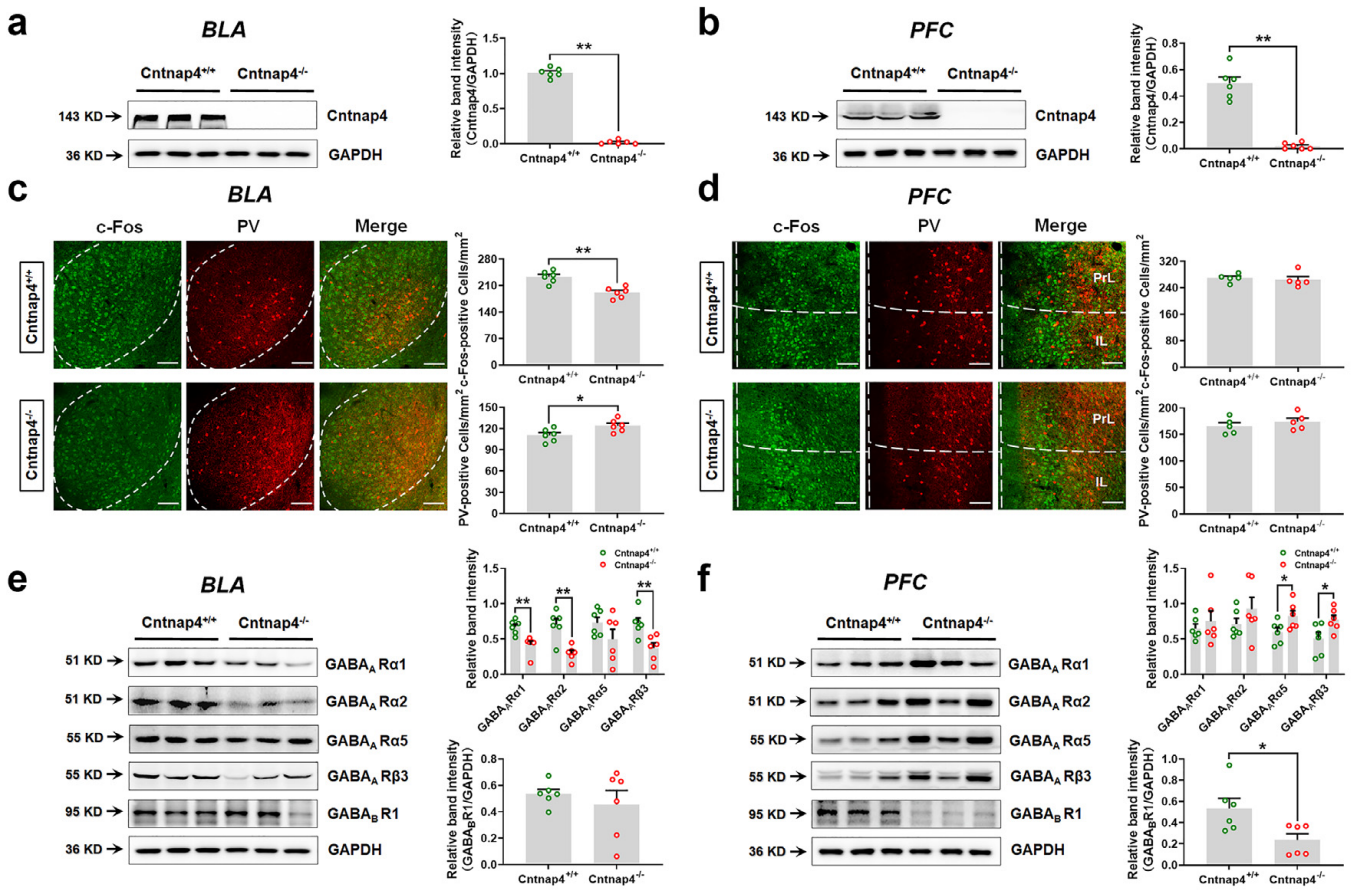
**GABA_A_ receptor expression is reduced in the BLA of male Cntnap4^−/−^ mice.** (a and b) Protein expression of Cntnap4 in the BLA and PFC of Cntnap4^+/+^ and Cntnap4^−/−^ mice. *n* = 6. (c and d) Immunofluorescent staining and quantitative analysis of PV+ and c-Fos + neurons in the BLA and PFC of Cntnap4^+/+^ and Cntnap4^−/−^ mice. n = 5–6. Scale bar = 200 μm. (e and f) Protein expression of GABA_A_Rα1, GABA_A_Rα2, GABA_A_Rα5, GABA_A_Rβ3 and GABA_B_R1 in the BLA and PFC of Cntnap4^+/+^ and Cntnap4^−/−^ mice. n = 6. Results are expressed as the mean ± SEM. ∗∗*p* < 0.01, ∗*p* < 0.05 vs. Cntnap4^+/+^ mice. Statistical significance was determined by Student's *t*-test.

**Supplementary Fig. 8:**
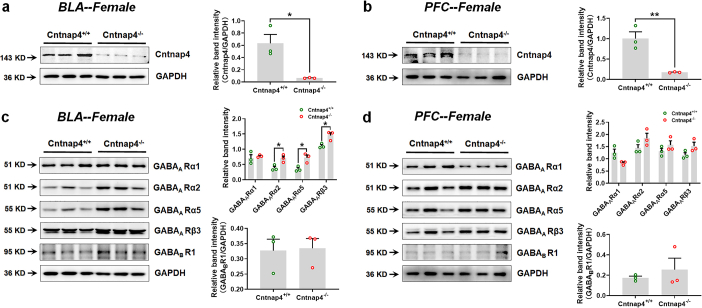


To further evaluate the molecular mechanisms of Cntnap4, we verified the ablation of *Cntnap4* (Fig. 5a, Student's *t* test, *t* = 33.14, df = 10, *p* < 0.0001; Fig. 5b, Student's *t* test, *t* = 9.739, df = 10, *p* < 0.0001) and to detect the immediate reaction of neurons, we then examined the numbers of immediate early gene c-Fos+ and palvalbumin (PV)+ neurons within 90 min in the BLA and PFC of male Cntnap4^−/−^ mice tested fear conditioning. Decreased c-Fos + neurons and increased PV+ neurons were observed in the BLA in male Cntnap4^−/−^ as compared with Cntnap4^+/+^ mice (Fig. 5c, Student's *t* test, c-Fos+: *t* = 4.766, df = 10, *p* = 0.0008; PV+: *t* = 2.637, df = 10, *p* = 0.0249). In contrast, neither c-Fos + nor PV+ neurons were changed in the PFC in male Cntnap4^−/−^ as compared with Cntnap4^+/+^ mice (Fig. 5d). Consistently, the expression of GABA_A_ type receptor family members GABA_A_Rα1, GABA_A_Rα2 and GABA_A_Rβ3 were decreased in the BLA in male Cntnap4^−/−^ mice (Fig. 5e, Student's *t* test, GABA_A_Rα1: *t* = 3.816, df = 10, *p* = 0.0034; GABA_A_Rα2: *t* = 4.419, df = 10, *p* = 0.0013; GABA_A_Rα5: *t* = 1.473, df = 10, *p* = 0.1716; GABA_A_Rβ3: *t* = 3.407, df = 10, *p* = 0.0067; GABA_B_R1: *t* = 0.7145, df = 10, *p* = 0.4913). Furthermore, the expression of GABA_A_Rα5 and GABA_A_Rβ3 were increased, while GABA_B_R1 was decreased in the PFC in male Cntnap4^−/−^ mice (Fig. 5f, Student's *t* test, GABA_A_Rα1: *t* = 0.6636, df = 10, *p* = 0.5219; GABA_A_Rα2: *t* = 1.173, df = 10, *p* = 0.2681; GABA_A_Rα5: *t* = 2.239, df = 10, *p* = 0.0491; GABA_A_Rβ3: *t* = 2.413, df = 10, *p* = 0.0365; GABA_B_R1: *t* = 2.706, df = 10, *p* = 0.0221). Somewhat different results were observed in female mice, for which Cntnap4 was verified to be ablated in the BLA and PFC and the expression levels of GABA_A_Rα2, GABA_A_Rα5 and GABA_A_Rβ3 were enhanced in the BLA in Cntnap4^−/−^ mice (Supplementary Fig. S8a, Student's *t* test, *t* = 3.872, df = 4, *p* = 0.0180; Supplementary Fig. S8b, Student's *t* test, *t* = 5.037, df = 4, *p* = 0.0073; Supplementary Fig. S8c, GABA_A_Rα1: *t* = 0.5698, df = 4, *p* = 0.5993; GABA_A_Rα2: *t* = 3.500, df = 4, *p* = 0.0249; GABA_A_Rα5: *t* = 3.727, df = 4, *p* = 0.0203; GABA_A_Rβ3: *t* = 3.607, df = 4, *p* = 0.0226; GABA_B_R1: *t* = 0.1606, df = 4, *p* = 0.8802). However, none of the GABA_A_ and GABA_B_ type receptors were changed in the PFC in female Cntnap4^−/−^ mice (Supplementary Fig. S8d). These results suggest that dynamic GABA receptor expression patterns and decreased amygdaloid GABA_A_ receptors in male Cntnap4^−/−^ mice may underlie the effects on GABAergic transmission, though the contribution of GABA_A_ receptors may be incrementally less in females. Collectively, the findings from electrophysiological recording, transcriptome analysis and molecular biology reveal that GABAergic transmission in male mice is disturbed upon *Cntnap4* deletion.

### Male Cntnap4^−/−^ mice have reduced levels of gut microbiota *Lactobacillus*

**Fig. 6:**
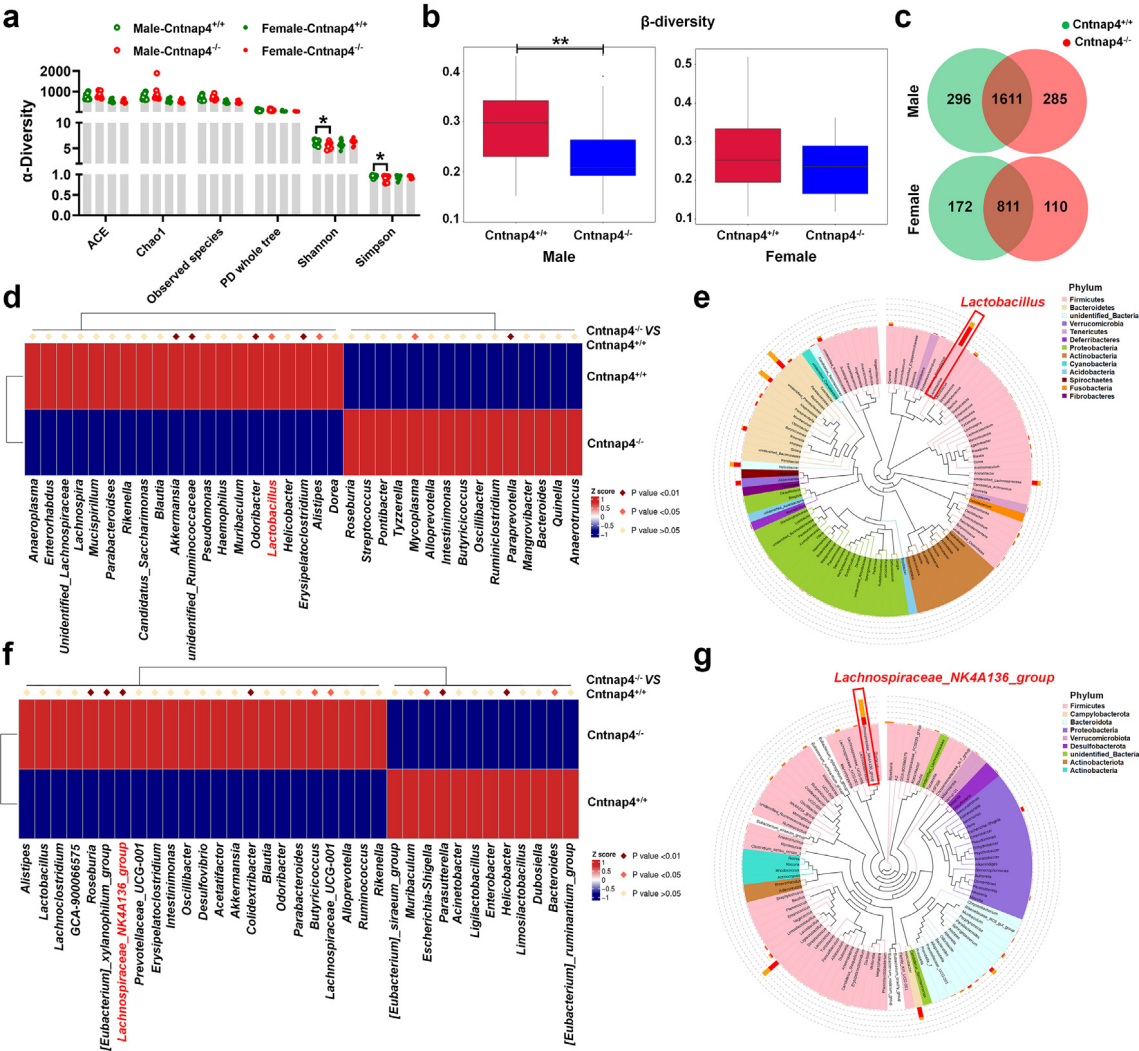
***Cntnap4*deficiency alters the gut microbiota composition.** (a and b) α-diversity (ACE, chao1, observed species, PD whole tree, Shannon and Simpson) and β-diversity in gut microbiota. (c) Venn diagram showing the overlap in microbiota composition between male and female Cntnap4^−/−^ and Cntnap4^+/+^ mice. (d) The differential gut microbiota between male Cntnap4^+/+^ and Cntnap4^−/−^ mice at the genus level. The abundance of *Lactobacillus* was significantly decreased at the genus level in Cntnap4^−/−^ mice (highlighted in red in d). (e) Phylogenetic relationships of species at the genus level in male Cntnap4^+/+^ and Cntnap4^−/−^ mice. *Lactobacillus* was framed. (f) The differential gut microbiota between female Cntnap4^+/+^ and Cntnap4^−/−^ mice at the genus level. The abundance of the *Lachnospiraceae_NK4A136_group* was significantly decreased at the genus level in Cntnap4^−/−^ mice (highlighted in red in f). (g) Phylogenetic relationships of species at the genus level in male Cntnap4^+/+^ and Cntnap4^−/−^ mice. *Lachnospiraceae_NK4A136_group* was framed. Results are expressed as the mean ± SEM. *n* = 11 and 8 for male Cntnap4^+/+^ and Cntnap4^−/−^ mice; *n* = 10 and 9 for female Cntnap4^+/+^ and Cntnap4^−/−^ mice. ∗∗*p* < 0.01, ∗*p* < 0.05 vs. Cntnap4^+/+^ mice. Statistical significance was determined by Student's *t*-test.

**Fig. 7:**
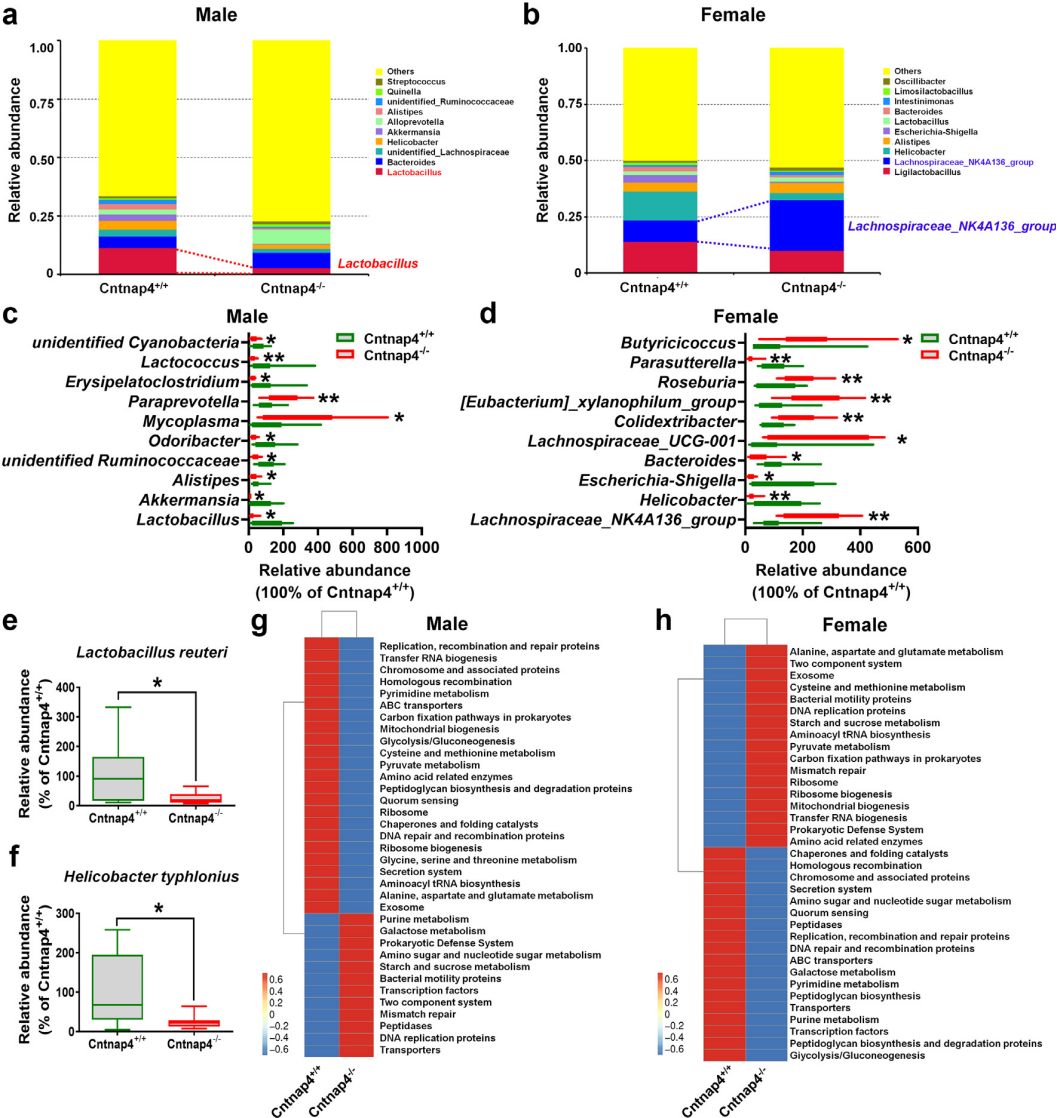
**The faecal*L. reuteri*level is decreased in male Cntnap4^−/−^ mice.** (a and b) Relative abundance of the top 10 differential gut microbiota at the genus level in male and female Cntnap4^+/+^ and Cntnap4^−/−^ mice. *Lactobacillus* was significantly decreased at the genus level in male Cntnap4^−/−^ mice (highlighted in red) compared with Cntnap4^+/+^ mice, and *Lachnospiraceae_NK4A136_group* was significantly decreased at the genus level in female Cntnap4^−/−^ mice (highlighted in blue) compared with Cntnap4^+/+^ mice. (c) The abundance of *Lactobacillus*, *Akkermansia*, *Alistipes*, *unidentified Ruminococcaceae*, *Odoribacter*, *Mycoplasma*, *Paraprevotella*, *Erysipelatoclostridium*, *Lactococcus*, and *unidentified Cyanobacteria* at the genus level in male Cntnap4^+/+^ and Cntnap4^−/−^ mice. (d) The abundance of *Lachnospiraceae_NK4A136_group*, *Helicobacter*, *Escherichia-Shigella*, *Bacteroides*, *Lachnospiraceae_UCG-001*, *Colidextribacter*, *[Eubacterium]_xylanophilum_group*, *Roseburia*, *Parasutterella*, and *Butyricicoccus* at the genus level in female Cntnap4^+/+^ and Cntnap4^−/−^ mice. (e and f) The abundance of *L. reuteri* and *Helicobacter typhlonius* at the species levels in male and female Cntnap4^+/+^ and Cntnap4^−/−^ mice. (g and h) The pathways enriched by the differential gut microbiota in male and female Cntnap4^+/+^ and Cntnap4^−/−^ mice. Results are expressed as the mean ± SEM. *n* = 11 and 8 for male Cntnap4^+/+^ and Cntnap4^−/−^ mice; *n* = 10 and 9 for female Cntnap4^+/+^ and Cntnap4^−/−^ mice. ∗∗*p* < 0.01, ∗*p* < 0.05 vs. Cntnap4^+/+^ mice. Statistical significance was determined by Student's *t*-test.

**Supplementary Fig. 9:**
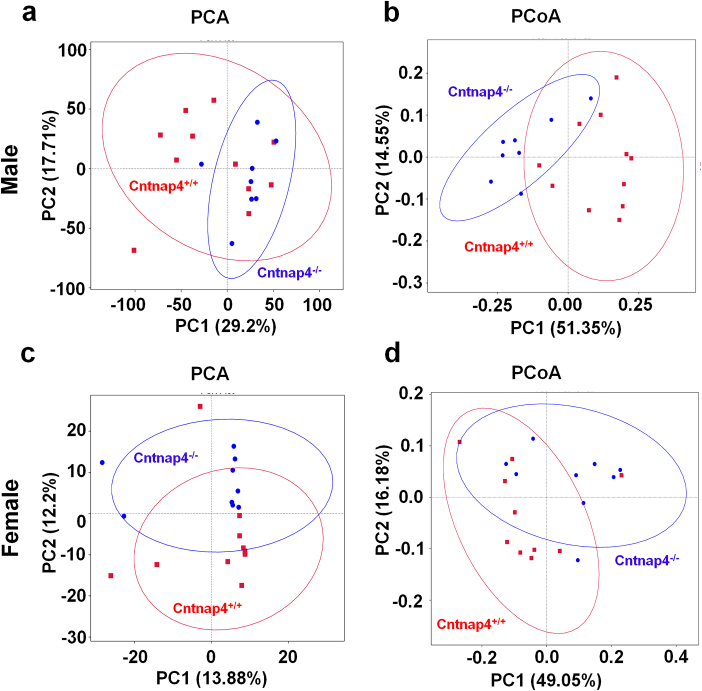


**Supplementary Fig. 10:**
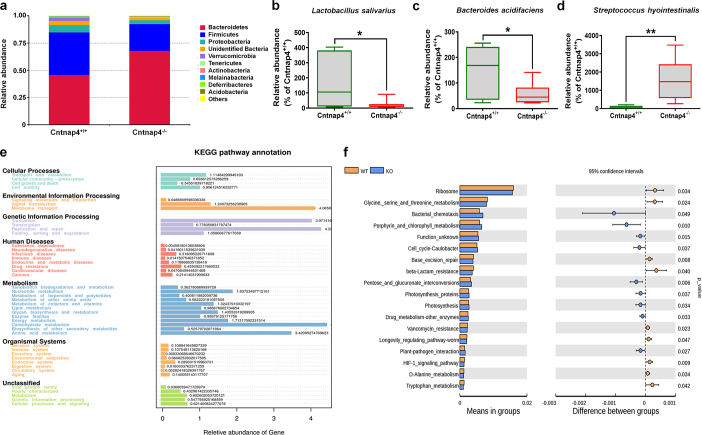


**Supplementary Fig. 11:**
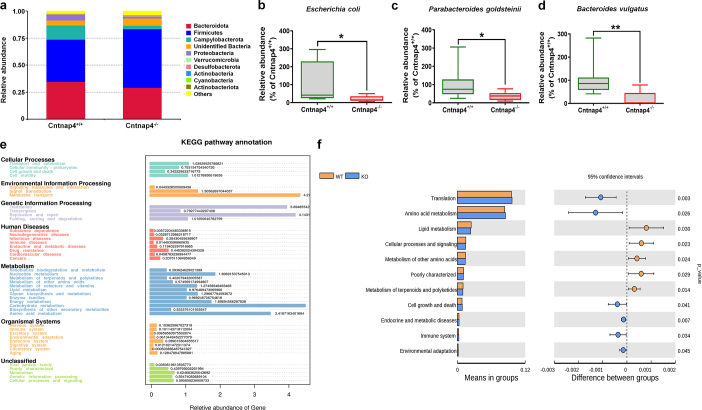


Altered gut microbiota is critical in modulating behaviour in ASD.^44^, ^45^, ^46^, ^47^ Therefore, we explored changes in the gut microbiota in male and female *Cntnap4* deficient mice. The levels of α-diversity (Shannon and Simpson) and β-diversity were decreased in male Cntnap4^−/−^ compared with Cntnap4^+/+^ mice (Fig. 6a and b, Student's *t* test, Shannon: *p* = 0.036; Simpson: *p* = 0.026; β-diversity: *p* = 0.004). In contrast, other α-diversity, including ACE, chao1, observed species and PD whole tree, showed no obvious differences between male or female Cntnap4^+/+^ and Cntnap4^−/−^ mice (Fig. 6a). Principal component analysis (PCA) and PCoA plots displayed the distribution of individuals in these two groups (Supplementary Fig. S9a-d), and Venn diagrams showed the overlap of differential microbiota compositions between male or female Cntnap4^+/+^ and Cntnap4^−/−^ mice (Fig. 6c). We evaluated the grouped relative microbiota abundance at the phylum level (Supplementary Fig. S10a and 11a), and we also showed the differential gut microbiota and phylogenetic relationships of species at the genus level in male and female Cntnap4^+/+^ and Cntnap4^−/−^ mice (Fig. 6d–g). Notably, *Lactobacillus* and *Lachnospiraceae_NK4A136_group* were the top hits in male and female Cntnap4^−/−^ compared with Cntnap4^+/+^ mice (Fig. 6d–g, Fig. 7a and b). We further listed the top 10 differential gut microbiota at the genus level in both male and female Cntnap4^+/+^ and Cntnap4^−/−^ mice (Fig. 7c, Student's *t* test, *Lactobacillus*: *p* = 0.0407, *Akkermansia*: *p* = 0.0191, *Alistipes*: *p* = 0.0269, *unidentified Ruminococcaceae*: *p* = 0.0158, *Odoribacter*: *p* = 0.0440, *Mycoplasma*: *p* = 0.0329, *Paraprevotella*: *p* = 0.0069, *Erysipelatoclostridium*: *p* = 0.0160, *Lactococcus*: *p* = 0.0075, *unidentified Cyanobacteria*: *p* = 0.0369; Fig. 7d, Student's *t* test, *Lachnospiraceae_NK4A136_group*: *p* = 0.0019, *Helicobacter*: *p* = 0.0079, *Escherichia-Shigella*: *p* = 0.0179, *Bacteroides*: *p* = 0.0299, *Lachnospiraceae_UCG-001*: *p* = 0.0489, *Colidextribacter*: *p* = 0.0060, *[Eubacterium]_xylanophilum_group*: *p* = 0.0030, *Roseburia*: *p* = 0.0079, *Parasutterella*, *p* = 0.0010, *Butyricicoccus*: *p* = 0.0229). Regarding male Cntnap4^−/−^ mice, *L. reuteri* and *L. salivarius* were identified as the major reduced *Lactobacillus* components at the species level (Fig. 7e, Student's *t* test, *L. reuteri*: *t* = 2.265, df = 17, *p* = 0.0369; Supplementary Fig. S10b, Student's *t* test, *L. salivarius*: *t* = 2.329, df = 17, *p* = 0.0325), while *Bacteroides acidifaciens* was found to be decreased and *Streptococcus hyointestinalis* was found to be increased (Supplementary Fig. S10c and d, Student's *t* test, *Bacteroides acidifaciens*: *t* = 2.406, df = 17, *p* = 0.0278; *S. hyointestinalis*: *t* = 4.456, df = 17, *p* = 0.0003). Regarding female Cntnap4^−/−^ mice, *Helicobacter typhlonius*, *Escherichia coli*, *Parabacteroides goldsteinii*, and *Bacteroides vulgatus* were identified the top hits of decreased microbiota at the species level (Fig. 7f, Student's *t* test, *H. typhlonius*: *t* = 2.548, df = 17, *p* = 0.0208; Supplementary Fig. S11b-d, Student's *t* test, *E. coli*: *t* = 2.118, df = 17, *p* = 0.0492; *P. goldsteinii*: *t* = 2.239, df = 17, *p* = 0.0388; *Bacteroides vulgatus*: *t* = 3.243, df = 17, *p* = 0.0048). Functional analysis suggested that the majority of the differential gut microbiota in male and female Cntnap4^−/−^ mice was enriched in “Environmental information processing”, “Genetic information processing” (such as transfer RNA biogenesis, replication, recombination and repair proteins, aminoacyl RNA biosynthesis) and “Metabolism” (such as mitochondrial biogenesis, pyruvate metabolism, glycolysis/gluconeogenesis) pathways (Fig. 7g and h, and Supplementary Fig. S10e and f, Fig. 11e and f). However, the differential gut microbiotas seemed not to participate in exactly same pathways, because half the pathways (17/34) were opposite in male and female Cntnap4^+/+^ and Cntnap4^−/−^ mice (Fig. 7g and h).

**Fig. 8:**
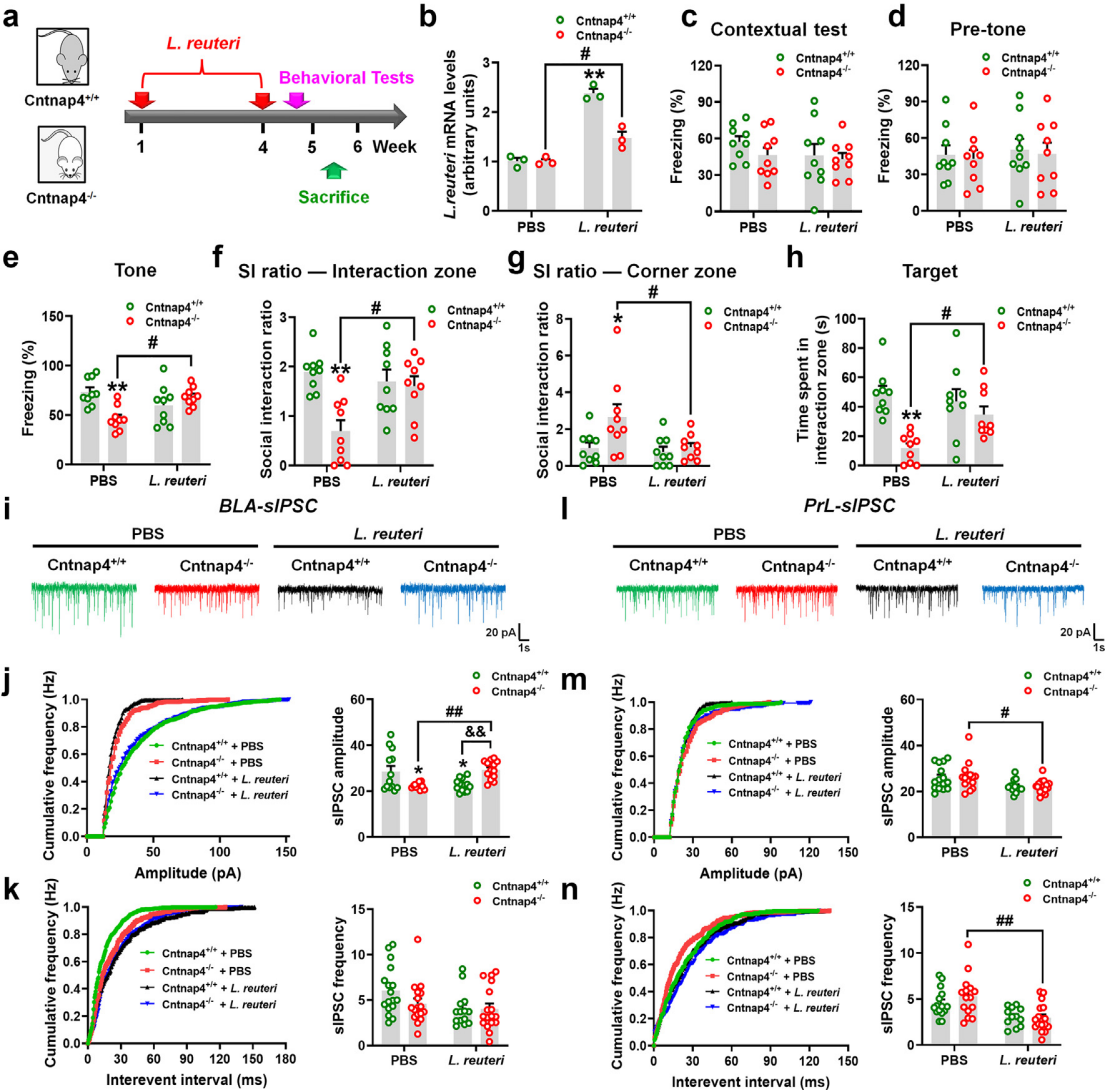
***L. reuteri*treatment rescues tone-cued fear conditioning and amygdaloid GABAergic transmission in male Cntnap4^−/−^ mice.** (a) Experimental design for *L. reuteri* administration in male Cntnap4^−/−^ mice. (b) The mRNA expressions of *L. reuteri* in the colon of male Cntnap4^+/+^ and Cntnap4^−/−^ mice treated with PBS or *L. reuteri*. *n* = 3 per group. *F*_1, 8_ = 26.34, *p* = 0.0009 for treatment–genotype interaction; *F*_1, 8_ = 109.3, *p* < 0.0001 for genotype; *F*_1, 8_ = 26.65, *p* = 0.0009 for treatment. (c and d) Freezing levels of male Cntnap4^+/+^ and Cntnap4^−/−^ mice treated with PBS or *L. reuteri* in the contextual test before the tone was applied. n = 9 per group. (c) *F*_1, 32_ = 0.3493, *p* = 0.5586 for treatment–genotype interaction; *F*_1, 32_ = 1.128, *p* = 0.2962 for genotype; *F*_1, 32_ = 1.114, *p* = 0.2990 for treatment. (d) *F*_1, 32_ = 4.575e-005, *p* = 0.9946 for treatment–genotype interaction; *F*_1, 32_ = 0.2412, *p* = 0.6267 for genotype; *F*_1, 32_ = 0.1664, *p* = 0.6860 for treatment. (e) *L. reuteri* increased the freezing time in male Cntnap4^−/−^ mice in tone-cued fear conditioning. *n* = 9 per group. *F*_1, 32_ = 14.04, *p* = 0.0007 for treatment–genotype interaction; *F*_1, 32_ = 0.7930, *p* = 0.3798 for genotype; *F*_1, 32_ = 3.848, *p* = 0.0586 for treatment. (f and g) SI ratio in the interaction and corner zone for male Cntnap4^+/+^ and Cntnap4^−/−^ mice treated with PBS or *L. reuteri*. *n* = 9 per group. (f) *F*_1, 32_ = 7.606, *p* = 0.0095 for treatment–genotype interaction; *F*_1, 32_ = 3.218, *p* = 0.0823 for genotype; *F*_1, 32_ = 10.35, *p* = 0.0030 for treatment. (g) *F*_1, 32_ = 2.868, *p* = 0.1000 for treatment–genotype interaction; *F*_1, 32_ = 4.727, *p* = 0.0372 for genotype; *F*_1, 32_ = 5.186, *p* = 0.0296 for treatment. (h) Time spent in the interaction zone for male Cntnap4^+/+^ and Cntnap4^−/−^ mice treated with PBS or *L. reuteri* with a target. *n* = 9 per group. *F*_1, 32_ = 5.741, *p* = 0.0226 for treatment–genotype interaction; *F*_1, 32_ = 2.256, *p* = 0.1429 for genotype; *F*_1, 32_ = 15.41, *p* = 0.0004 for treatment. (i and l) Representative traces of GABA receptor-mediated sIPSCs in the BLA and PrL. All sIPSCs were recorded at a holding potential of −70 mV. (j and m) Cumulative frequency plots (left) and quantitative analysis (right) of the amplitude of GABA receptor-mediated sIPSCs in the BLA and PrL. (j) *F*_1, 47_ = 21.13, *p* < 0.0001 for treatment–genotype interaction; *F*_1, 47_ = 0.1655, *p* = 0.6860 for genotype; *F*_1, 47_ = 0.02387, *p* = 0.8779 for treatment. (m) *F*_1, 55_ = 0.03771, *p* = 0.8467 for treatment–genotype interaction; *F*_1, 55_ = 11.57, *p* = 0.0013 for genotype; *F*_1, 55_ = 0.06133, *p* = 0.8053 for treatment. (k and n) Cumulative frequency plots of the interevent interval (left) and quantitative analysis of the frequency of GABA receptor-mediated sIPSCs (right) in the BLA and PrL. (k) *F*_1, 61_ = 1.630, *p* = 0.2065 for treatment–genotype interaction; *F*_1, 61_ = 5.101, *p* = 0.0275 for genotype; *F*_1, 61_ = 1.412, *p* = 0.2393 for treatment. (n) *F*_1, 55_ = 1.070, *p* = 0.3054 for treatment–genotype interaction; *F*_1, 55_ = 19.41, *p* < 0.0001 for genotype; *F*_1, 55_ = 0.5539, *p* = 0.4599 for treatment. 11–17 slices from *n* = 4 mice per group. Results are expressed as the mean ± SEM. ∗∗*p* < 0.01, ∗*p* < 0.05 vs. Cntnap4^+/+^ + PBS mice; ^##^*p* < 0.01, ^#^*p* < 0.05 vs. Cntnap4^−/−^ + PBS mice; ^&&^*p* < 0.01 vs. Cntnap4^+/+^ + *L. reuteri* mice. Statistical significance was determined by two-way ANOVA + Bonferroni's multiple comparisons test.

**Fig. 9:**
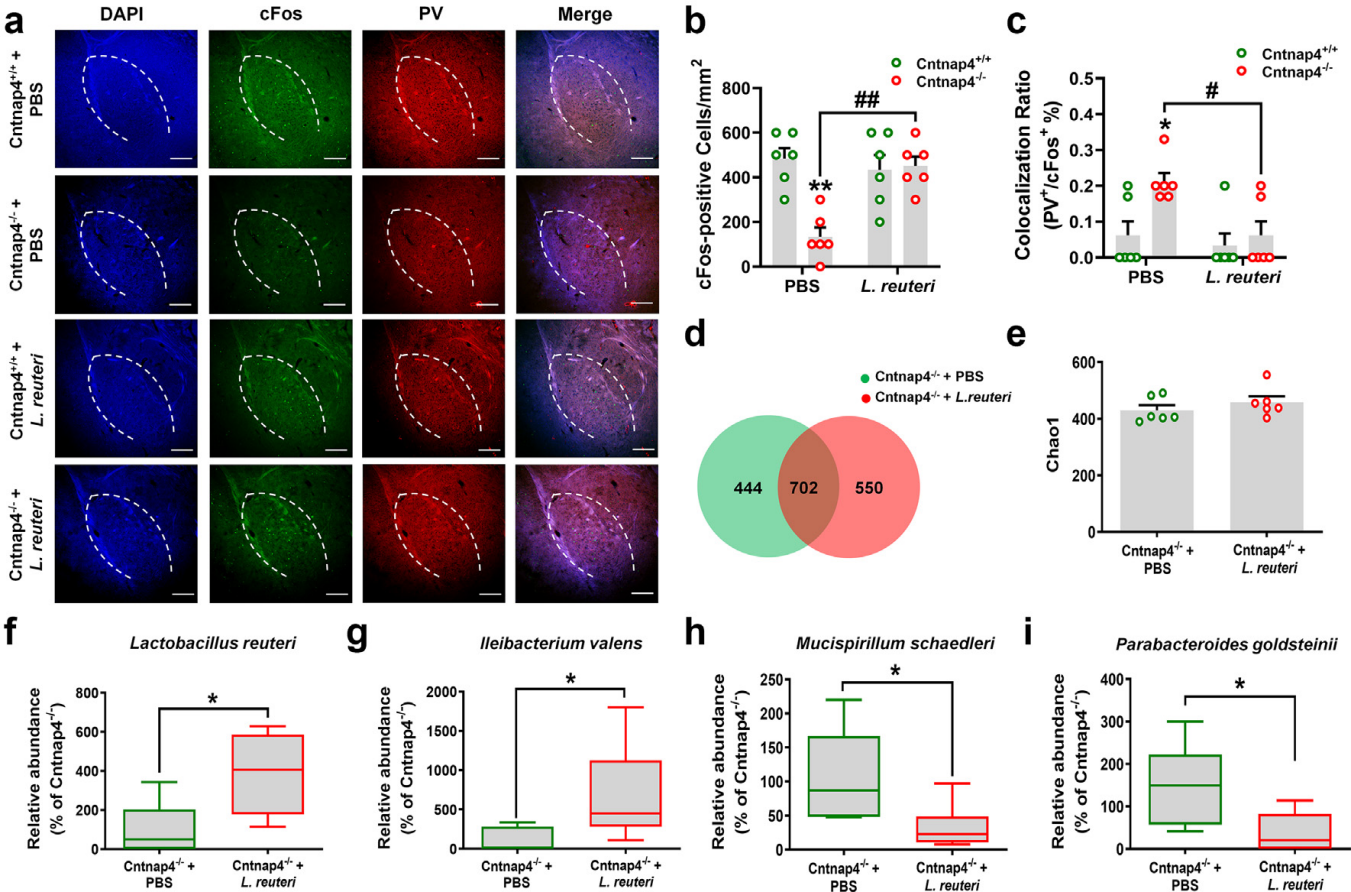
***L. reuteri*treatment increases c-Fos + neurons in the BLA in male Cntnap4^−/−^ mice.** (a–c) Immunofluorescent staining and quantitative analysis of c-Fos + neurons and the PV+/c-Fos + colocalization ratio in the BLA of Cntnap4^+/+^ and Cntnap4^−/−^ mice treated with PBS or *L. reuteri*. (b) *F*_1, 20_ = 13.01, *p* = 0.0018 for treatment–genotype interaction; *F*_1, 20_ = 6.882, *p* = 0.0163 for genotype; *F*_1, 20_ = 10.75, *p* = 0.0038 for treatment. (c) *F*_1, 20_ = 3.097, *p* = 0.0937 for treatment–genotype interaction; *F*_1, 20_ = 6.654, *p* = 0.0179 for genotype; *F*_1, 20_ = 6.654, *p* = 0.0179 for treatment. *n* = 6 per group. Scale bar = 500 μm. (d) Venn diagram showing the overlap in the differential microbiota composition between Cntnap4^−/−^ + PBS and Cntnap4^−/−^ + *L. reuteri* mice. (e) α-diversity (Chao1) in gut microbiota between Cntnap4^−/−^ + PBS and Cntnap4^−/−^ + *L. reuteri* mice. (f–i) Relative abundance of faecal *L. reuteri*, *Ileibacterium valens*, *Mucispirillum schaedleri* and *Parabacteroides goldsteinii* at the species level between Cntnap4^−/−^ + PBS and Cntnap4^−/−^ + *L. reuteri* mice. n = 6 per group. Results are expressed as the mean ± SEM. ∗∗*p* < 0.01, ∗*p* < 0.05 vs. Cntnap4^+/+^ + PBS mice (for b and c) or Cntnap4^−/−^ + PBS mice (for e-i); ^##^*p* < 0.01, ^#^*p* < 0.05 vs. Cntnap4^−/−^ + PBS mice. Statistical significance was determined by two-way ANOVA + Bonferroni's multiple comparisons test (for b and c) and Student's *t*-test (for e–i).

**Fig. 10:**
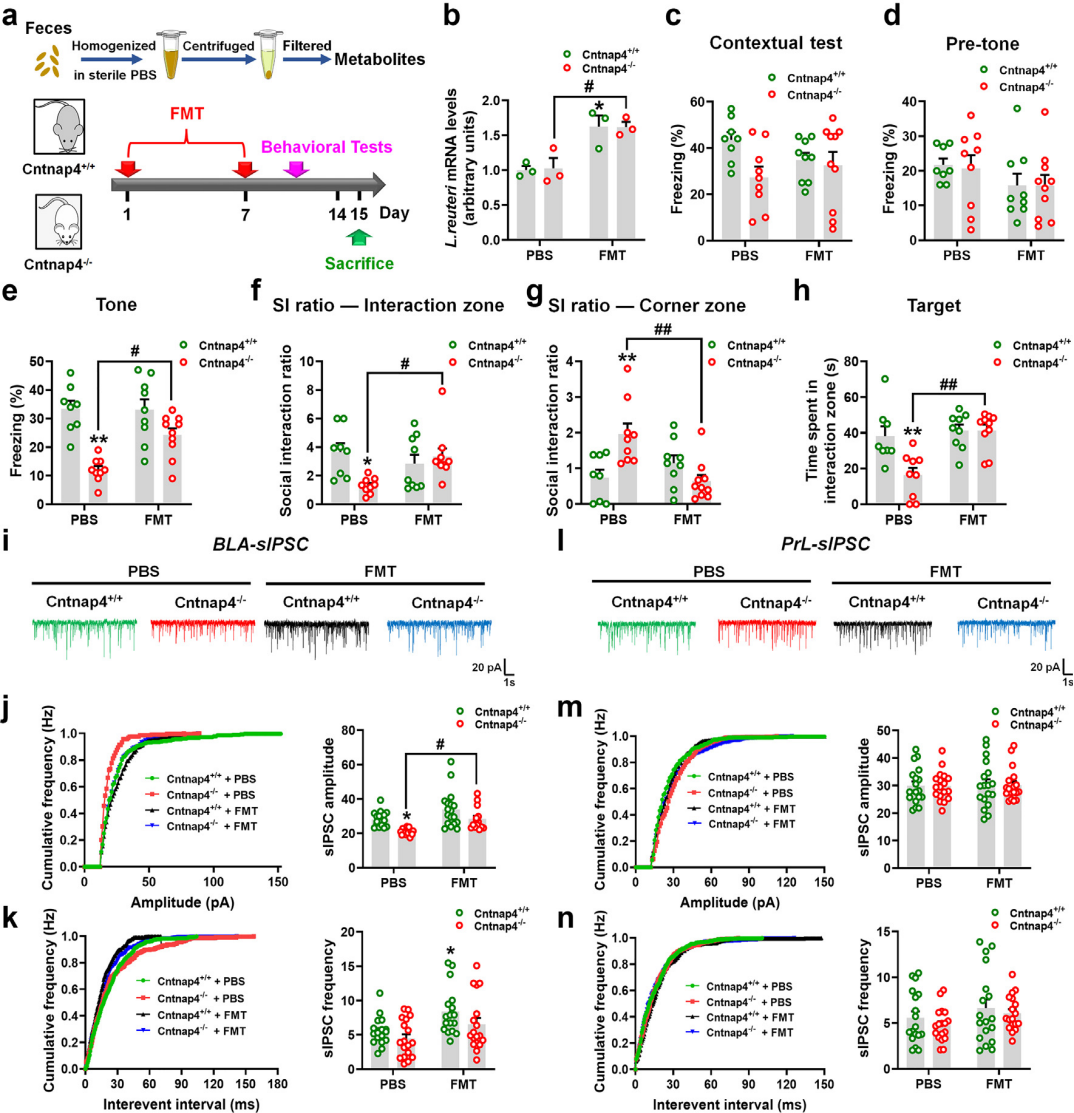
**Transplantation of the faecal microbiota rescues tone-cued fear conditioning and amygdaloid GABAergic transmission in male Cntnap4^−/−^ mice.** (a) Experimental design for FMT administration in male Cntnap4^−/−^ mice. (b) The mRNA expressions of *L. reuteri* in the colon of male Cntnap4^+/+^ and Cntnap4^−/−^ mice treated with PBS or FMT. *n* = 3 per group. *F*_1, 8_ = 0.0173, *p* = 0.8985 for treatment–genotype interaction; *F*_1, 8_ = 26.39, *p* = 0.0009 for genotype; *F*_1, 8_ = 0.0058, *p* = 0.9413 for treatment. (c and d) Freezing levels of male Cntnap4^+/+^ and Cntnap4^−/−^ mice upon PBS treatment or FMT in the contextual test before the tone was applied. (c) *F*_1, 32_ = 2.380, *p* = 0.1327 for treatment–genotype interaction; *F*_1, 32_ = 0.1382, *p* = 0.7125 for genotype; *F*_1, 32_ = 4.210, *p* = 0.0485 for treatment. (d) *F*_1, 32_ = 0.02411, *p* = 0.8776 for treatment–genotype interaction; *F*_1, 32_ = 2.853, *p* = 0.1009 for genotype; *F*_1, 32_ = 0.03214, *p* = 0.8588 for treatment. (e) FMT increased the freezing time in male Cntnap4^−/−^ mice in tone-cued fear conditioning. *F*_1, 32_ = 5.313, *p* = 0.0278 for treatment–genotype interaction; *F*_1, 32_ = 4.698, *p* = 0.0377 for genotype; *F*_1, 32_ = 30.59, *p* < 0.0001 for treatment. (f and g) SI ratio in the interaction and corner zone for male Cntnap4^+/+^ and Cntnap4^−/−^ mice upon PBS treatment or FMT. (f) *F*_1, 32_ = 7.487, *p* = 0.0101 for treatment–genotype interaction; *F*_1, 32_ = 1.397, *p* = 0.2460 for genotype; *F*_1, 32_ = 3.108, *p* = 0.0875 for treatment. (g) *F*_1, 32_ = 13.73, *p* = 0.0008 for treatment–genotype interaction; *F*_1, 32_ = 3.800, *p* = 0.0600 for genotype; F_1, 32_ = 2.329, *p* = 0.1368 for treatment. (h) Time spent in the interaction zone for male Cntnap4^+/+^ and Cntnap4^−/−^ mice upon PBS treatment or FMT with a target. *F*_1, 32_ = 7.320, *p* = 0.0108 for treatment–genotype interaction; *F*_1, 32_ = 12.05, *p* = 0.0015 for genotype; *F*_1, 32_ = 7.170, *p* = 0.0116 for treatment. *n* = 8, 9, 9, 10 for Cntnap4^+/+^ + PBS, Cntnap4^+/+^ + FMT, Cntnap4^−/−^ + PBS, Cntnap4^−/−^ + FMT, respectively. (i and l) Representative traces of GABA receptor-mediated sIPSCs in the BLA and PrL. All sIPSCs were recorded at a holding potential of −70 mV. (j and m) Cumulative frequency plots (left) and quantitative analysis (right) of the amplitude of GABA receptor-mediated sIPSCs in the BLA and PrL. (j) *F*_1, 60_ = 0.2793, *p* = 0.5991 for treatment–genotype interaction; *F*_1, 60_ = 14.49, *p* = 0.0003 for genotype; *F*_1, 60_ = 13.58, *p* = 0.0005 for treatment. (m) *F*_1, 68_ = 0.0006, *p* = 0.9803 for treatment–genotype interaction; *F*_1, 68_ = 0.05369, *p* = 0.8175 for genotype; *F*_1, 68_ = 0.003415, *p* = 0.9536 for treatment. (k and n) Cumulative frequency plots of the interevent interval (left) and quantitative analysis of the frequency of GABA receptor-mediated sIPSCs (right) in the BLA and PrL. (k) *F*_1, 67_ = 0.2678, *p* = 0.6065 for treatment–genotype interaction; *F*_1, 67_ = 11.18, *p* = 0.0014 for genotype; *F*_1, 67_ = 3.871, *p* = 0.0533 for treatment. (n) *F*_1, 68_ = 0.03963, *p* = 0.8428 for treatment–genotype interaction; *F*_1, 68_ = 3.075, *p* = 0.0840 for genotype; *F*_1, 68_ = 1.094, *p* = 0.2993 for treatment. 14–18 slices from *n* = 4 mice per group. Results are expressed as the mean ± SEM. ∗∗*p* < 0.01, ∗*p* < 0.05 vs. Cntnap4^+/+^ + PBS mice; ^##^*p* < 0.01, ^#^*p* < 0.05 vs. Cntnap4^−/−^ + PBS mice. Statistical significance was determined by two-way ANOVA + Bonferroni's multiple comparisons test.

**Fig. 11:**
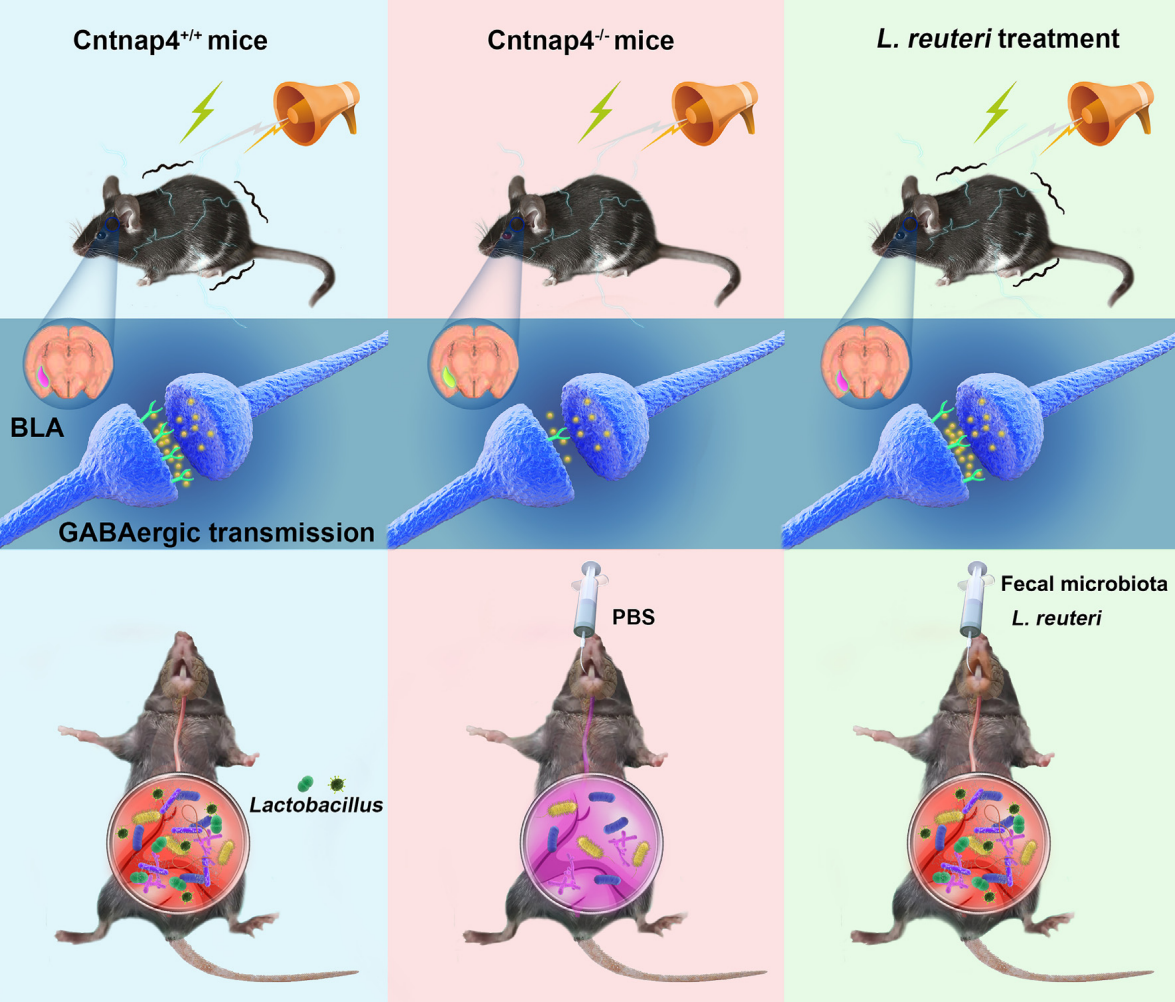
**Illustration design of this study.** In the normal condition, *Lactobacillus* maintains the processing of fear memory via regulating GABAergic transmission in BLA. Upon *Cntnap4* deficiency, male mice manifest *Lactobacillus* (especially *L. reuteri*) loss and dysfunction of GABAergic transmission in BLA. Ultimately, *Lactobacillus reuteri* supplementation or faecal microbiota transplantation restores the tone-cued fear memory possibly via amelioration of amygdala GABAergic transmission.

These results suggest that *Lactobacillus* is the predominant form of reduced gut microbiota in male Cntnap4^−/−^ mice, and that its reduced levels may be responsible for the impaired fear conditioning and GABAergic transmission in male Cntnap4^−/−^ mice.

### Treatment with *L. reuteri* rescues fear conditioning and GABAergic transmission in male Cntnap4^−/−^ mice

**Supplementary Fig. 12:**
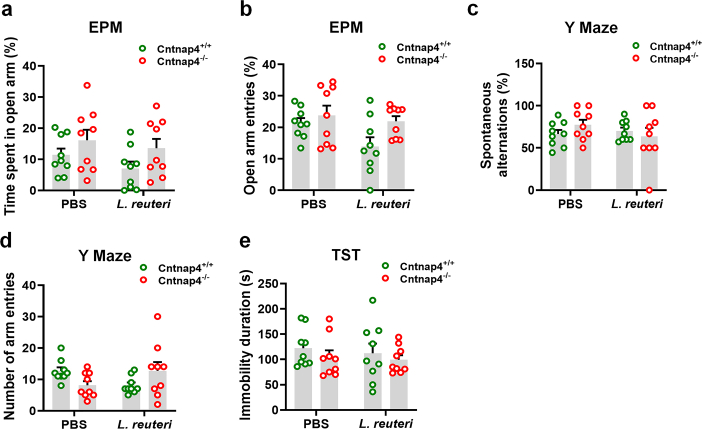


**Supplementary Fig. 13:**
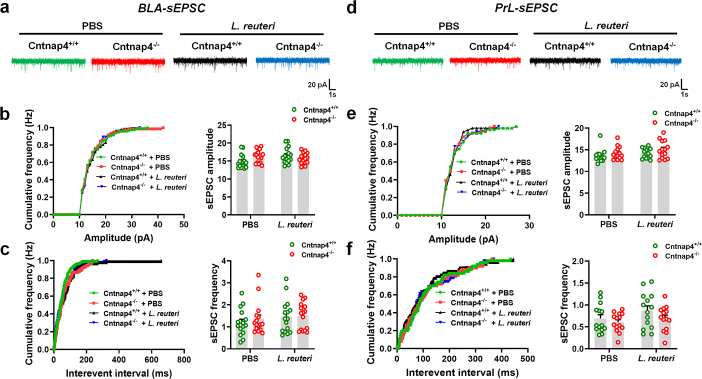


Given that *L. reuteri* has been reported to relieve ASD-related behavioural abnormalities,^57^ we wondered whether it could rescue impaired fear conditioning and GABAergic transmission in male Cntnap4^−/−^ mice. To evaluate this possibility, we fed male Cntnap4^−/−^ mice with PBS or *L. reuteri* for 4 weeks (Fig. 8a). We confirmed the colonization of the gastrointestinal tract by *L. reuteri* by examining its mRNA expression in the colon (Fig. 8b, *p* < 0.0001 for *L. reuteri* vs. PBS treatment in Cntnap4^+/+^ mice, *p* = 0.0229 for *L. reuteri* vs. PBS treatment in Cntnap4^−/−^ mice). There were no obvious effects on the contextual test and pre-tone phase (Fig. 8c and d); however, *L. reuteri* reversed tone-cued fear conditioning in Cntnap4^−/−^ mice (Fig. 8e, *p* = 0.0017 for Cntnap4^+/+^ mice vs. Cntnap4^−/−^ mice upon PBS treatment, *p* = 0.0128 for *L. reuteri* vs. PBS treatment in Cntnap4^−/−^ mice). *L. reuteri* treatment also increased the social interaction ratio in the interaction zone and decreased it in the corner zone, alongside enhanced duration in the interaction zone under the target condition (Fig. 8f–h, *p* = 0.0010, 0.0399, and 0.0005 for Cntnap4^+/+^ mice vs. Cntnap4^−/−^ mice upon PBS treatment; *p* = 0.0149, 0.0472, and 0.0449 for *L. reuteri* vs. PBS treatment in Cntnap4^−/−^ mice, respectively). Furthermore, *L. reuteri* did not affect the behavioural performance in the EPM, Y maze and TST (Supplementary Fig. S12a–e). Remarkably, however, *L. reuteri* reversed the decreased sIPSC amplitude in the BLA in Cntnap4^−/−^ mice (Fig. 8i and j, *p* = 0.0191 for Cntnap4^+/+^ mice vs. Cntnap4^−/−^ mice upon PBS treatment, *p* = 0.0186 for *L. reuteri* vs. PBS treatment in Cntnap4^+/+^ mice, *p* = 0.0066 for *L. reuteri* vs. PBS treatment in Cntnap4^−/−^ mice, *p* = 0.0060 for Cntnap4^+/+^ mice vs. Cntnap4^−/−^ mice upon *L. reuteri* treatment). *L. reuteri* treatment also showed no obvious effects on the sIPSC frequency in the BLA in male Cntnap4^−/−^ mice (Fig. 8k) but decreased the sIPSC amplitude and frequency in the PrL in male Cntnap4^−/−^ mice (Fig. 8l–n, *p* = 0.0483 and 0.0010 for *L. reuteri* vs. PBS treatment in Cntnap4^−/−^ mice, respectively). Moreover, no obvious changes were observed in the sEPSC amplitude and frequency in the BLA and PrL of Cntnap4^−/−^ mice upon *L. reuteri* treatment (Supplementary Fig. S13a–f).

**Supplementary Fig. 14:**
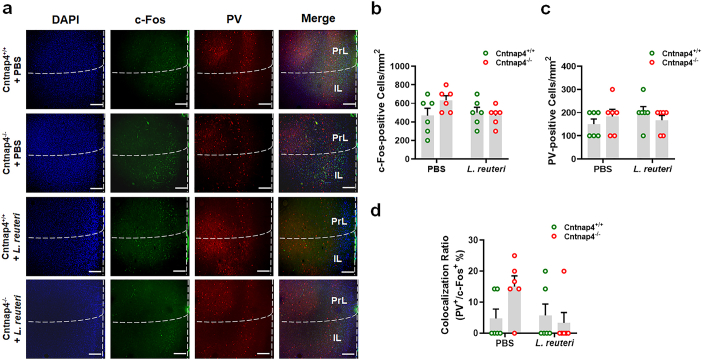


**Supplementary Fig. 15:**
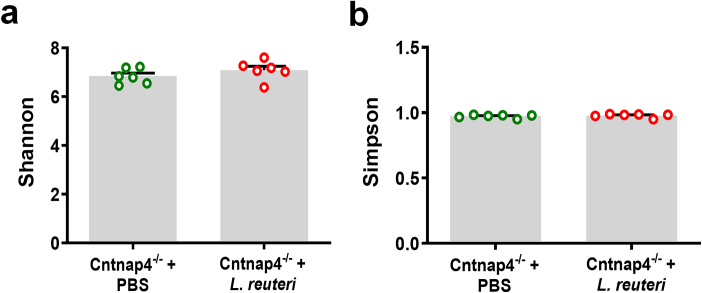


For additional evidence of the effects of *L. reuteri* treatment, we examined the neurons in male Cntnap4^−/−^ mice. *L. reuteri* treatment increased c-Fos + neurons and decreased PV+/c-Fos + colocalized neurons in the BLA (Fig. 9a–c, Fig. 9b: *p* = 0.0005 for Cntnap4^+/+^ mice vs. Cntnap4^−/−^ mice upon PBS treatment, *p* = 0.0014 for *L. reuteri* vs. PBS treatment in Cntnap4^−/−^ mice; Fig. 9c: *p* = 0.0285 for Cntnap4^+/+^ mice vs. Cntnap4^−/−^ mice upon PBS treatment, *p* = 0.0285 for *L. reuteri* vs. PBS treatment in Cntnap4^−/−^ mice). On the other hand, there were no obvious effects on the PV+ and c-Fos + neurons in the PFC in male Cntnap4^−/−^ mice (Supplementary Fig. S14a–d). Furthermore, *L. reuteri* treatment did not alter the levels of α-diversity, including Chao1, Shannon and Simpson diversity (Fig. 9d and e, Supplementary Fig. S15a and b). However, it significantly increased the relative abundance of *L. reuteri*, suggesting that *L. reuteri* treatment mediates a change in the microbial composition of the host (Fig. 9f, Student's *t* test, *t* = 2.740, df = 10, *p* = 0.0208). Moreover, *L. reuteri* treatment also increased the faecal *Ileibacterium valens* level and decreased the *Mucispirillum schaedleri* and *P. goldsteinii* levels in male Cntnap4^−/−^ mice (Fig. 9g–i, *I. valens*: Student's *t* test, *t* = 2.242, df = 10, *p* = 0.0488; *M. schaedleri*: Student's *t* test, *t* = 2.391, df = 10, *p* = 0.0379; *P. goldsteinii*: Student's *t* test, t = 2.547, df = 10, *p* = 0.0290). Collectively, these observations reveal that *L. reuteri* treatment rescues impaired tone-cued fear conditioning and GABAergic synaptic transmission in male Cntnap4^−/−^ mice.

### Transplantation of the faecal microbiota from Cntnap4^+/+^ mice restores the fear memory and GABAergic transmission in male Cntnap4^−/−^ mice

**Supplementary Fig. 16:**
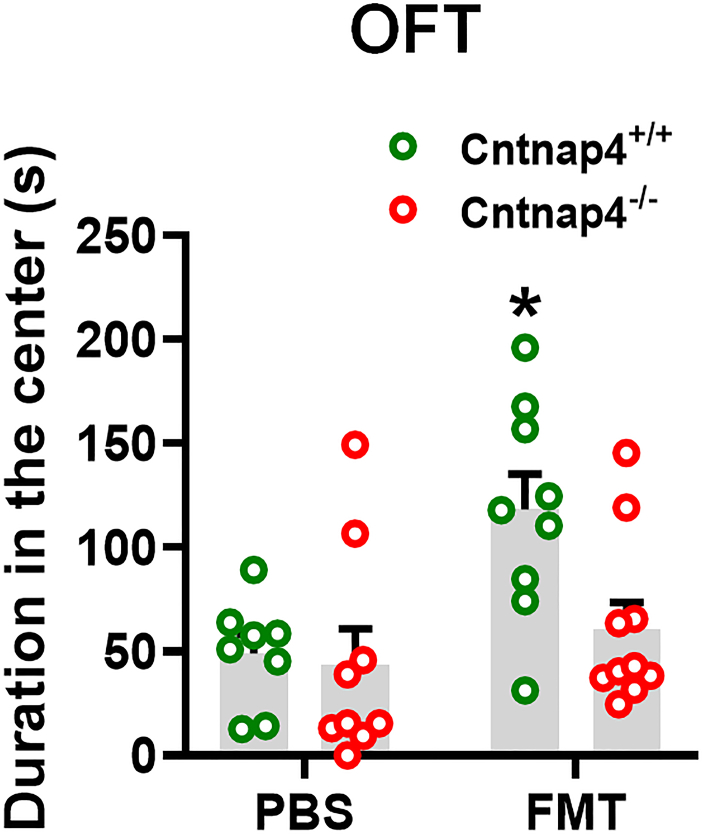


Since microbiota transfer therapy is a potential tool for treatment of ASD,^64^ we then performed FMT experiments to evaluate if the microbes from Cntnap4^+/+^ mice could rescue the fear memory and GABAergic transmission in male Cntnap4^−/−^ mice. Male Cntnap4^+/+^ and Cntnap4^−/−^ mice were gavaged the faecal microbiota from 8-week-old male wild type C57BL/6J mice for 1 week (Fig. 10a). We found FMT also increased the colonization of *L. reuteri* in the colon (Fig. 10b, *p* = 0.0242 for FMT vs. PBS treatment in Cntnap4^+/+^ mice, *p* = 0.0312 for FMT vs. PBS treatment in Cntnap4^−/−^ mice). Here, FMT increased the time male Cntnap4^+/+^ mice spent in the centre zone of the open field (Supplementary Fig. S16, *p* = 0.0130 for FMT vs. PBS treatment in Cntnap4^+/+^ mice). During the fear conditioning test, there were no obvious effects on the contextual test or pre-tone phase (Fig. 10c and d), while FMT rescued tone-cued fear conditioning in Cntnap4^−/−^ mice (Fig. 10e, *p* < 0.0001 for Cntnap4^+/+^ mice vs. Cntnap4^−/−^ mice upon PBS treatment, *p* = 0.0136 for FMT vs. PBS treatment in Cntnap4^−/−^ mice). FMT also enhanced the social interaction ratio in the interaction zone (Fig. 10f, *p* = 0.0201 for Cntnap4^+/+^ mice vs. Cntnap4^−/−^ mice upon PBS treatment, *p* = 0.0361 for FMT vs. PBS treatment in Cntnap4^−/−^ mice) and decreased it in the corner zone (Fig. 10g, *p* = 0.0056 for Cntnap4^+/+^ mice vs. Cntnap4^−/−^ mice upon PBS treatment, *p* = 0.0014 for FMT vs. PBS treatment in Cntnap4^−/−^ mice). The duration of Cntnap4^−/−^ mice in the interaction zone was reversed upon FMT intervention with the target (Fig. 10h, *p* = 0.0042 for Cntnap4^+/+^ mice vs. Cntnap4^−/−^ mice upon PBS treatment, *p* = 0.0005 for FMT vs. PBS treatment in Cntnap4^−/−^ mice). Consistently, FMT also reversed the decreased the sIPSC amplitude in the BLA in Cntnap4^−/−^ mice (Fig. 10i and j, *p* = 0.0211 for Cntnap4^+/+^ mice vs. Cntnap4^−/−^ mice upon PBS treatment, *p* = 0.0268 for FMT vs. PBS treatment in Cntnap4^−/−^ mice). Furthermore, FMT increased the sIPSC frequency in the BLA in male Cntnap4^+/+^ mice (Fig. 10k, *p* = 0.0414 for FMT vs. PBS treatment in Cntnap4^+/+^ mice). FMT showed no obvious effects on the sIPSC amplitude and frequency in the PrL in male Cntnap4^−/−^ mice (Fig. 10l–n). Thus, FMT also restores the impaired fear conditioning and GABAergic synaptic transmission in male Cntnap4^−/−^ mice.

## Discussion

The gut–brain axis adds a new dimension to autism therapy, as emerging evidence indicates that gut microbes skillfully modulate social and emotional behaviours. In the present study, we reveal that social behaviours, fear conditioning and GABAergic synaptic transmission are impaired in male Cntnap4^−/−^ mice. Based on an altered gut microbiota community after *Cntnap4* ablation, we provide evidence that *L. reuteri* treatment or faecal microbiota transplantation rescues impaired tone-cued fear conditioning and GABAergic transmission in male Cntnap4^−/−^ mice.

Previously, Cntnap4 was found to be localized presynaptically, and its deficiency was shown to reduce the output of cortical PV+ GABAergic basket cells.^28^ Cntnap4^−/−^ mice manifest sensory-motor gating and over-grooming, which are core symptoms of ASD.^28^ In this study, we report that male Cntnap4^−/−^ mice lack a preference for novel social partners and display a social novelty recognition deficit. Furthermore, *Cntnap4* deficiency also affects tone-cued fear memory processing in male mice. The amygdala is critical for input and processing of emotional memories, especially including fear memory acquisition, consolidation and extinction,^11^^,^^65^, ^66^, ^67^ and amygdala projection neurons are primarily innervated by GABAergic interneurons. Amygdaloid GABAergic mechanisms, such as neuregulin 1-ErbB4 and TMEM16B/ANO2 signalling, contribute significantly to fear memory processing.^12^^,^^68^^,^^69^ As Cntnap4 is expressed in interneurons in the amygdala,^30^ our results support a role for Cntnap4 in GABAergic transmission and tone-cued fear conditioning because nearly 90% of mature cortical PV+ interneurons express Cntnap4,^28^ and amygdala pyramidal neurons are regulated by local GABAergic interneurons (∼10% of the neuronal population). *Cntnap4* loss reduces GABA release and affects GABAergic receptors expressed in amygdala pyramidal neurons in the BLA. Accordingly, the sIPSC amplitude but not frequency was decreased in male Cntnap4^−/−^ mice. Additionally, RNA-seq and molecular biological results showed disturbed GABA_A_ type receptor expression upon *Cntnap4* deficiency. Thus, our results may provide new evidence for *Cntnap4* loss participating in amygdala GABAergic transmission chaos in ASD. In this study, we observed that *Cntnap4* deficiency induced decreased GABA-related gene or receptor expression and reduced GABAergic transmission, alongside increased PV+ cells in the BLA. To test the role of GAD67 in GABAergic neuron, previously, Fujihara et al. generated *PV-Cre*, *GAD67*^*flox/flox*^ (homozygous) and *PV-Cre*, and *GAD67*^*flox/+*^ (heterozygous) mice.^70^ They found that homozygous *GAD67* deletion in PV neurons leads to a reduction in GAD67 and GABA immunoreactivities and substantial increases in PV immunoreactivity.^70^ Since GAD-67-mediated GABA synthesis shapes the PV-synaptic innervations during the maturation of inhibitory circuits,^71^ they speculated that this effect may contribute to the excitation of pyramidal neurons and induce compensatory responses in homozygous *PV-Cre*; *GAD67*^*flox/flox*^ mice.^70^ Because almost all Cntnap4+ cells within the somatosensory cortex are GAD67 positive and almost all mature PV cells (∼94%) express Cntnap4,^28^ the increased populations of PV+ neurons mediated by *Cntnap4* deficiency may be also a compensatory change in response to the decrease in GABA-related gene or receptor expression.

Although the majority of neurons (at least 85%) in the BLA projecting to the prefrontal cortex or ventral striatum are excitatory neurons,^72^ we did not observe apparent changes in sEPSC amplitude and frequency in the BLA and PFC of Cntnap4^−/−^ mice. On the one hand, given that Cntnap4 is mainly expressed in interneurons, its ablation exerts less effect on excitatory synaptic transmission. On the other hand, these results may indicate that the BLA-PFC projection is unaltered upon *Cntnap4* knockout. Nevertheless, we still need conditional *Cntnap4* knockout in PV+ neurons to draw this conclusion. Furthermore, we acknowledge that we did not observe the social deficits, impaired fear conditioning, together with disturbed GABAergic transmission in female Cntnap4^−/−^ mice. Mechanistically, unlike the downregulated GABAergic transmission in male Cntnap4^−/−^ mice, we confirmed the increased GABAergic receptor expression in the amygdala of female Cntnap4^−/−^ mice alongside reduced estradiol and oestrogen receptor levels. Oestrogen is critical in regulating neuronal activity and animal behaviour associated with ASD.^73^ Previously, oestrogen receptors were found to be colocalized in GABAergic neurons,^74^^,^^75^ and there are dynamic results regarding oestrogen regulating GABAergic synapse in the brain. In the medial preoptic area and other hypothalamus areas, oestrogen acts to increase GABA release and reuptake as well as GABA_A_ receptor expression.^76^, ^77^, ^78^ However, in the amygdala and hippocampus, oestrogen acts to suppress GABAergic transmission by inhibiting GABA_A_ receptors expression.^77^^,^^79^ We conclude that *Cntnap4* ablation decreases estradiol levels, and thus increases GABAergic synapses, which may compromise the dysfunctional GABAergic transmission observed in male Cntnap4^−/−^ mice. Nevertheless, female Cntnap4^−/−^ mice also display hair loss due to overgrooming and show impaired spontaneous alterations in the Y maze test, suggesting that their working memory may be damaged. Indeed, *Cntnap4* deletion may impair hippocampal learning and memory in female mice, given that we and other groups have revealed that Cntnap4 plays an important role in aging-related diseases, such as PD and AD.^36^^,^^38^ Thus, the effects of hormonal influences and neural circuit development on GABAergic transmission result in the sex differences in processing fear memory.^80^

Notably, we demonstrated that *L. reuteri* treatment rescues impaired tone-cued fear conditioning and GABAergic transmission in male Cntnap4^−/−^ mice. Recently, manipulating gut microbiota has been shown to provide an attractive approach in treating neurological diseases.^81^ Previously, ASD patients have been identified to harbour altered gut microorganisms and serious gastrointestinal problems,^44^^,^^45^ and many meaningful studies suggest that microbiota treatment is a promising intervention for ASD patients.^47^^,^^64^ Although there seems to be a shift in the general microbial composition between male Cntnap4^+/+^ and Cntnap4^−/−^ mice, we focused on *L. reuteri* in this study. First, we identified *Lactobacillus* is the predominant form of reduced gut microbiota in male Cntnap4^−/−^ mice, and both *L. reuteri* and *L. salivarius* were decreased in male Cntnap4^−/−^ mice. Meanwhile, the content of *L. reuteri* was much more abundant than that of *L. salivarius*. Second, *L. reuteri* has been reported to recover social deficits in ASD models,^57^^,^^82^ and clinical trials are also on the way to evaluate *L. reuteri* interventions in ASD patients.^83^ Although *L. salivarius* is also a probiotic candidate, it is reported to influence the host immune system and possess anti-inflammatory properties.^84^ Moreover, *L. salivarius* has been revealed to prevent inflammation in different disease models, such as intestinal and skin inflammation.^85^^,^^86^ Herein, we report that *L. reuteri* administration restores tone-cued fear conditioning, and it also enhances GABAergic transmission in male Cntnap4^−/−^ mice. Given that FMT is an emerging approach for the treatment of ASD,^47^^,^^64^ our data also support that FMT rescues the fear conditioning and GABAergic transmission in male Cntnap4^−/−^ mice. Our results showed that Cntnap4^−/−^ mice treated with *L. reuteri* were improved in the behavioural tests and brain activity, the *L. reuteri* colonization in Cntnap4^−/−^ mice is actually much lower than the Cntnap4^+/+^. Considering together the transfer of fecal materials from Cntnap4^+/+^ to Cntnap4^−/−^ mice also rescued the behaviours, it is more convincing that the entire shift of microbial composition benefits the Cntnap4^−/−^ mice more than *L. reuteri* itself. Our study broadens the potential role of microbiota in ASD therapy and provide insights for its potential mechanisms of action.

In summary, we provide evidence that *Cntnap4* deficiency impairs social behaviours and tone-cued fear conditioning. Altered GABAergic synaptic transmission in BLA and disturbed gut microbiota composition underlie these behavioural abnormalities. Remarkably, *L. reuteri* administration or faecal microbiota transplantation rescues impaired tone-cued fear conditioning and GABAergic transmission in male Cntnap4^−/−^ mice. Taken together, our findings highlight the potential of *L. reuteri* treatment as a new approach to ameliorate ASD (Fig. 11).

## Contributors

Y.L.Z. designed the experiments, supervised the project, and wrote the manuscript. W.L.Z., and M.R.Z. performed the Western blotting experiments and analysed the data. J.H., and Q.L.Y. performed the electrophysiological experiments. X.D.S. analysed the electrophysiological data. F.G., and L.Y.D. performed the immunostaining. J.W.G., R.F.M., and S.H.Z. helped with animal surgery and behavioural experiments as well as data analysis. W.L.Z., and Y.L.Z. have directly accessed and verified the underlying data reported in the manuscript. All authors read and approved the final manuscript.

## Data sharing

All data needed to evaluate the conclusions in the paper are presented in the paper and/or the Supplementary Materials. RNA-seq data used in this study are available under GEO: GSE208542 for male Cntnap4^+/+^ and Cntnap4^−/−^ mice and GSE208397 for female Cntnap4^+/+^ and Cntnap4^−/−^ mice. Additional data related to this paper may be requested from the corresponding author.

## Declaration of interests

The authors declare no competing interests.

## Appendix A Supplementary data

Supplementary Table S1

Captions for Supplementary Figures

Reagent Validation

Supplemental Western blots
