# Supplementary material for: Lactobacillus reuteri normalizes altered fear memory in male Cntnap4 knockout mice
Source: eBioMedicine. 2022 Nov 15;86:104323. doi: 10.1016/j.ebiom.2022.104323 (PMC9672961; doi:10.1016/j.ebiom.2022.104323)
Supplement: Supplementary Table S1 [file mmc1.docx]

**Supplementary Table 1. Primer sequences used for qRT-PCR.**

| Mice genes | Primer sequence (5′-3′) |
| --- | --- |
| *Car2* | F: TATGATAAAGCTGCGTCCAAGA  R: CAATCTGTAGGAGTCACTGAGG |
| *Gabra1* | F: TTCACAAGAATTTTGGACCGAC  R: CCTTCCAACTTTGACGGAAAAA |
| *Gabrb2* | F: TACTCAGCACGCTTGAGATAAA  R: CAATGGCATTCACATCAGTCAA |
| *Gabrg1* | F: ATGGGCTATTTCACGATTCAGA  R: GTGATACCCAGGGATGTTCTAG |
| *Gabrg2* | F: CGGTTGAATAGCAATATGGTGG  R: GTGTAGAGAACTCGACCATCAT |
| *Kif5b* | F: TCCAGAAGGCATGGGAATTATT  R: TTCATCTGGACTACACACGAAA |
| *Slc38a2* | F: TCCGTTCACCTCCTCCTCAAGAC  R: CCAGCCAGACCGTATGCCTTATG |
| *Gls2* | F: CATGCTGCCTCGACTTGGTGAC  R: GCCGTGGTGAACTTGTGGATAGG |
| *Gls* | F: CACTCAAATCTACAGGATTGCG  R: CCAGACTGCTTTTTAGCACTTT |
| *Slc38a1* | F: CATCATGGGCAGTGGAATCTTGGG  R: CAGCAGTGTCACCGAAGTCAGAAG |
| *Gabrr2* | F: ATACAGCATGAGGATTACGGTC  R: AGATCTTCATCTGTGTACGCAT |
| *Gabrg3* | F: TGAGACCGGACATTGGAATAAA  R: ACTTGGTGAATCTCGAAGTCTT |
| *Esr1* | F: CTACTACCTGGAGAACGAGC  R: GCGTCGATTGTCAGAATTAGAC |
| *Esr2* | F: CTTCGCAAGTGTTACGAAGTAG  R: GCACTTCTCTGTCTTCGTACTA |
| *Pgr* | F: TAGTCTCGCCTATACCGATCTC  R: CTTCCCTATGAGTGGCTTCTAC |
| *Ar* | F: TAAAGACATTTTGAACGAGGCC  R: GTCAGATATGGTTGAATTGCCC |
| *Gapdh* | F: ACGGGAAGCTCACTGGCATGGCCTT  R: CATGAGGTCCACCACCCTGTTGCTG |
| *L. reuteri* | F: GAAGATCAGTCGCAYTGGCCCAA  R: TCCATTGTGGCCGATCAG |
| *General bacteria* | F: ACTCCTACGGGAGGCAGCAG  R: ATTACCGCGGCTGCTGG |

The primer sequences of *Car2*, *Gabra1*, *Gabrb2*, *Gabrg1*, *Gabrg2, Kif5b*, *Slc38a2*, *Gls2*, *Gls*, *Slc38a1*, *Gabrr2*, *Gabrg3*, *Esr1*, *Esr2*, *Pgr*, *Ar*, *Gapdh*, *L. reuteri*, *and General bacteria*.
