# Supplementary material for: Lactobacillus reuteri normalizes altered fear memory in male Cntnap4 knockout mice
Source: eBioMedicine. 2022 Nov 15;86:104323. doi: 10.1016/j.ebiom.2022.104323 (PMC9672961; doi:10.1016/j.ebiom.2022.104323)
Supplement: Captions for Supplementary Figures [file mmc2.docx]

**Supplementary Figure Legends**

**Supplementary Figure 1. Behavioral tests of Cntnap4^-/-^ mice.** (a) Travel tracings of mice in the open-field. (b) The number of entries into the center zone of mice in the open field test. *F*_1, 37_ = 3.338, *p* = 0.0758 for sex-genotype interaction; *F*_1, 37_ = 0.001925, *p* = 0.9652 for genotype; *F*_1, 37_ = 1.486, *p* = 0.2306 for sex. (c) Duration in the center zone in open field. *F*_1, 37_ = 2.964e-005, *p* = 0.9957 for sex-genotype interaction; *F*_1, 37_ = 0.5327, *p* = 0.4701 for genotype; *F*_1, 37_ = 0.4281, *p* = 0.5170 for sex. (d) Spontaneous alterations (%) were used to evaluate working memory in the Y maze test. *F*_1, 37_ = 3.572, *p* = 0.0666 for sex-genotype interaction; *F*_1, 37_ = 1.933, *p* = 0.1727 for genotype; *F*_1, 37_ = 6.124, *p* = 0.0180 for sex. (e) Time spent in the open arm in the elevated plus maze (EPM) test. *F*_1, 37_ = 0.03569, *p* = 0.8512 for sex-genotype interaction; *F*_1, 37_ = 1.488, *p* = 0.2303 for genotype; *F*_1, 37_ = 2.481, *p* = 0.1237 for sex. (f) Immobility time in the tail suspension test. *F*_1, 37_ = 0.1557, *p* = 0.6955 for sex-genotype interaction; *F*_1, 37_ = 0.1705, *p* = 0.6821 for genotype; *F*_1, 37_ = 0.3573, *p* = 0.5537 for sex. Results are expressed as the mean ± SEM. n = 11, 10, 12, 8 for male and female Cntnap4^+/+^ and Cntnap4^-/-^ mice, respectively. Statistical significance was determined by two-way ANOVA + Bonferroni’s multiple comparisons test.

**Supplementary Figure 2. Impact of *Cntnap4* deletion on excitatory transmission.** (a and d) Representative traces of AMPA receptor-mediated sEPSCs in the BLA and PrL. All sIPSCs were recorded at a holding potential of −70 mV. (b and e) Cumulative frequency plots of the amplitude (left) and quantitative analysis of the amplitude of AMPA receptor-mediated sEPSCs (right) in the BLA and PrL. (b) *F*_1, 72_ = 0.008264, *p* = 0.9278 for sex-genotype interaction; *F*_1, 72_ = 6.874, *p* = 0.0107 for genotype; *F*_1, 72_ = 5.831, *p* = 0.0107 for sex. (e) *F*_1, 72_ = 2.277, *p* = 0.1357 for sex-genotype interaction; *F*_1, 72_ = 0.1390, *p* = 0.7104 for genotype; *F*_1, 72_ = 4.232, *p* = 0.0433 for sex. (c and f) Cumulative frequency plots of the interevent interval (left) and quantitative analysis of the frequency of AMPA receptor-mediated sEPSCs (right) in the BLA and PrL. (c) *F*_1, 72_ = 3.457, *p* = 0.0671 for sex-genotype interaction; *F*_1, 72_ = 12.19, *p* = 0.0008 for genotype; *F*_1, 72_ = 15.88, *p* = 0.0002 for sex. (f) *F*_1, 72_ = 0.6944, *p* = 0.4074 for sex-genotype interaction; *F*_1, 72_ = 0.0123, *p* = 0.9120 for genotype; *F*_1, 72_ = 7.562, *p* = 0.0075 for sex. Results are expressed as the mean ± SEM. 16–22 slices from n = 4 mice per group. ^**^*p* < 0.01 vs. Cntnap4^+/+^ mice. Statistical significance was determined by two-way ANOVA + Bonferroni’s multiple comparisons test.

**Supplementary Figure 3. Transcriptome analysis indicators.** Fpkm density distribution in the BLA of male Cntnap4^+/+^ and Cntnap4^-/-^ mice.

**Supplementary Figure 4. KEGG pathways enriched in downregulated DEGs.** Downregulated DEGs between male Cntnap4^+/+^ and Cntnap4^-/-^ mice enriched in KEGG pathways.

**Supplementary Figure 5. GO pathways enriched in upregulated DEGs.** Upregulated DEGs between male Cntnap4^+/+^ and Cntnap4^-/-^ mice enriched in GO pathways.

**Supplementary Figure 6. KEGG pathways enriched by upregulated DEGs.** Upregulated DEGs between male Cntnap4^+/+^ and Cntnap4^-/-^ mice enriched in KEGG pathways.

**Supplementary Figure 7. Transcriptome analysis indicators.** Gene expression distribution (a) and Fpkm density distribution (b) in the BLA of female Cntnap4^+/+^ and Cntnap4^-/-^ mice.

**Supplementary Figure 8. Impact of *Cntnap4* deficiency on the expressions of GABA receptors in female Cntnap4^-/-^ mice.** (a and b) Protein expression of Cntnap4 in the BLA and PFC of Cntnap4^+/+^ and Cntnap4^-/-^ mice. n = 3. (c and d) Protein expression of GABA_A_Rα1, GABA_A_Rα2, GABA_A_Rα5, GABA_A_Rβ3 and GABA_B_R1 in the BLA and PFC of Cntnap4^+/+^ and Cntnap4^-/-^ mice. n = 3. Results are expressed as the mean ± SEM. ^**^*p* < 0.01, ^*^*p* < 0.05 vs. Cntnap4^+/+^ mice. Statistical significance was determined by Student’s *t*-test.

**S****upplementary Figure 9. The α-diversity of gut microbiota in male and female Cntnap4^-/-^ mice.** (a and b) PCA and PCoA plots indicate the distribution of individuals in male Cntnap4^+/+^ and Cntnap4^-/-^ mice. (c and d) PCA and PCoA plots indicate the distribution of individuals in female Cntnap4^+/+^ and Cntnap4^-/-^ mice. Results are expressed as the mean ± SEM. n = 11 and 8 for male Cntnap4^+/+^ and Cntnap4^-/-^ mice; n = 10 and 9 for female Cntnap4^+/+^ and Cntnap4^-/-^ mice.

**Supplementary Figure 10. Differential gut microbiota and signaling pathway****s in male Cntnap4^-/-^ mice.** (a) Relative abundance of the top 10 differential gut microbiota at the phylum level. (b-d) Relative abundance of *Lactobacillus salivarius*, *Bacteroides acidifaciens* and *Streptococcus hyointestinalis* at the species level in male Cntnap4^-/-^ mice. (e) KEGG pathway annotation. (f) Potential pathways enriched by differential gut microbiota between male Cntnap4^+/+^ and Cntnap4^-/-^ mice. Results are expressed as the mean ± SEM. n = 11 and 8 for male Cntnap4^+/+^ and Cntnap4^-/-^ mice. ^*^*p* < 0.05 vs. Cntnap4^+/+^ mice. Statistical significance was determined by Student’s *t*-test.

**Supplementary Figure 11. Differential gut microbiota and signaling pathways in female Cntnap4^-/-^ mice.** (a) Relative abundance of the top 10 differential gut microbiota at the phylum level. (b-d) Relative abundance of *Escherichia coli*, *Parabacteroides goldsteinii* and *Bacteroides vulgatus* at the species level in female Cntnap4^-/-^ mice. (e) KEGG pathway annotation. (f) Potential pathways enriched by differential gut microbiota between female Cntnap4^+/+^ and Cntnap4^-/-^ mice. Results are expressed as the mean ± SEM. n = 10 and 9 for female Cntnap4^+/+^ and Cntnap4^-/-^ mice. ^*^*p* < 0.05 vs. Cntnap4^+/+^ mice. Statistical significance was determined by Student’s *t*-test.

**Supplementary Figure 12. Effects of *L. reuteri* treatment on the behavioral performance in Cntnap4^-/-^ mice.** (a and b) Time spent in the open arm and open arm entries in the EPM test. (a) *F*_1, 32_ = 0.1096, *p* = 0.7427 for treatment-genotype interaction; *F*_1, 32_ = 1.603, *p* = 0.2146 for genotype; *F*_1, 32_ = 4.265, *p* = 0.0471 for treatment. (b) *F*_1, 32_ = 1.336, *p* = 0.2562 for treatment-genotype interaction; *F*_1, 32_ = 3.788, *p* = 0.0605 for genotype; *F*_1, 32_ = 4.683, *p* = 0.0380 for treatment. (c and d) Spontaneous alterations (%) and number of arm entries in the Y maze test. (c) *F*_1, 32_ = 1.673, *p* = 0.2051 for treatment-genotype interaction; *F*_1, 32_ = 0.5657, *p* = 0.4575 for genotype; *F*_1, 32_ = 0.1038, *p* = 0.7494 for treatment. (d) *F*_1, 32_ = 6.919, *p* = 0.0130 for treatment-genotype interaction; *F*_1, 32_ = 0.000, *p* > 0.9999 for genotype; *F*_1, 32_ = 0.000, *p* > 0.9999 for treatment. (e) Immobility time in the tail suspension test. *F*_1, 32_ = 0.02662, *p* = 0.8714 for treatment-genotype interaction; *F*_1, 32_ = 0.3060, *p* = 0.5840 for genotype; *F*_1, 32_ = 1.163, *p* = 0.2889 for treatment. n = 9 per group. Results are expressed as the mean ± SEM. Statistical significance was determined by two-way ANOVA + Bonferroni’s multiple comparisons test.

**Supplementary Figure 13. Impact of *L. reuteri* treatment on the excitatory transmission in Cntnap4^-/-^ mice.** (a and d) Representative traces of AMPA receptor-mediated sEPSCs in the BLA and PrL. All sEPSCs were recorded at a holding potential of −70 mV. (b and e) Cumulative frequency plots of the amplitude (left) and quantitative analysis of the amplitude of AMPA receptor-mediated sEPSCs (right) in the BLA and PrL. (b) *F*_1, 60_ = 6.406, *p* = 0.0140 for treatment-genotype interaction; *F*_1, 60_ = 0.8839, *p* = 0.3509 for genotype; *F*_1, 60_ = 0.5010, *p* = 0.4818 for treatment. (e) *F*_1, 52_ = 0.1096, *p* = 0.7420 for treatment-genotype interaction; *F*_1, 52_ = 2.590, *p* = 0.1136 for genotype; *F*_1, 52_ = 2.896, *p* = 0.0948 for treatment. (c and f) Cumulative frequency plots of the interevent interval (left) and quantitative analysis of the frequency of AMPA receptor-mediated sEPSCs (right) in the BLA and PrL. (c) *F*_1, 60_ = 0.1244, *p* = 0.7256 for treatment-genotype interaction; *F*_1, 60_ = 1.609, *p* = 0.2096 for genotype; *F*_1, 60_ = 0.3828, *p* = 0.5385 for treatment. (f) *F*_1, 52_ = 0.3164, *p* = 0.5762 for treatment-genotype interaction; *F*_1, 52_ = 2.521, *p* = 0.1184 for genotype; *F*_1, 52_ = 2.521, *p* = 0.1184 for treatment. 14–16 slices from n = 4 mice per group. Results are expressed as the mean ± SEM. Statistical significance was determined by two-way ANOVA + Bonferroni’s multiple comparisons test.

**Supplementary Figure 14. The number of PV+ and c-Fos+ neurons in the PFC upon *L. reuteri* treatment in male Cntnap4^-/-^ mice.** (a) Immunofluorescent staining of PV+ and c-Fos+ neurons in the PFC of Cntnap4^+/+^ and Cntnap4^-/-^ mice treated with PBS or *L. reuteri*. Scale bar = 500 μm. (b-d) Quantitative analysis of c-Fos+ neurons, PV+ neurons, and the PV+/c-Fos+ colocalization ratio in the PFC of Cntnap4^+/+^ and Cntnap4^-/-^ mice treated with PBS or *L. reuteri*. (b) *F*_1, 20_ = 2.857, *p* = 0.1065 for treatment-genotype interaction; *F*_1, 20_ = 1.270, *p* = 0.2731 for genotype; *F*_1, 20_ = 1.270, *p* = 0.2731 for treatment. (c) *F*_1, 20_ = 1.739, *p* = 0.2022 for treatment-genotype interaction; *F*_1, 20_ = 0.4348, *p* = 0.5172 for genotype; *F*_1, 20_ = 0.000, *p* > 0.9999 for treatment. (d) *F*_1, 20_ = 3.516, *p* = 0.0755 for treatment-genotype interaction; *F*_1, 20_ = 2.538, *p* = 0.1268 for genotype; *F*_1, 20_ = 1.368, *p* = 0.2559 for treatment. n = 6 per group. Results are expressed as the mean ± SEM. Statistical significance was determined by two-way ANOVA + Bonferroni’s multiple comparisons test.

**Supplementary Figure 15. The α-diversity of gut microbiota in Cntnap4^-/-^ mice treated with *L. reuteri*.** The α-diversity (Shannon and Simpson) of gut microbiota was analyzed in Cntnap4^-/-^ mice treated with PBS or *L. reuteri*. n = 6 per group. Results are expressed as the mean ± SEM. Statistical significance was determined by Student’s *t*-test.

**Supplementary Figure 16. The effect of FMT on the behavioral performance in the OFT.** Duration in the centre zone of the open field. *F*_1, 32_ = 3.206, *p* = 0.0828 for treatment–genotype interaction; *F*_1, 32_ = 8.789, *p* = 0.0057 for genotype; *F*_1, 32_ = 4.626, *p* = 0.0391 for treatment. *n* = 8, 9, 9, 10 for Cntnap4^+/+^ + PBS, Cntnap4^+/+^ + FMT, Cntnap4^-/-^ + PBS, Cntnap4^-/-^ + FMT, respectively. Results are expressed as the mean ± SEM. ^*^*p* < 0.05 vs. Cntnap4^+/+^ + PBS mice. Statistical significance was determined by two-way ANOVA + Bonferroni’s multiple comparisons test.
