# Supplementary material for: Lactobacillus reuteri normalizes altered fear memory in male Cntnap4 knockout mice
Source: eBioMedicine. 2022 Nov 15;86:104323. doi: 10.1016/j.ebiom.2022.104323 (PMC9672961; doi:10.1016/j.ebiom.2022.104323)
Supplement: Reagent Validation [file mmc3.pdf]

Antibody validation

Antibodies used in this study are all commercial antibodies, and we provide RRID tags for the antibodies. If the RRID tags are not available, we provide the relevant references for their applications.

1. Anti-GABA$_A$Rα1 (Cat# 06-868; Research Resource Identifiers [RRID]: AB_310272) antibody was purchased from Millipore (Billerica, MA, USA).

In the original manuscript, 3108661 is the lot number and we correct it in the revised manuscript.

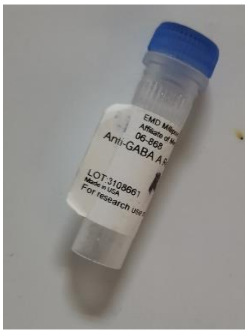

2. Anti-GABA$_A$Rα2 (Cat# ab193311; RRID: AB_2890213) antibody was purchased from Abcam (Cambridge, MA, USA).

3. Anti-GABA$_A$$\alpha$5 (Cat# sc-393921; RRID not available), GABA$_A$Rβ3 (Cat# sc-376252; RRID: AB_11012142), GABA$_B$R1 (Cat# sc-166408; RRID: AB_2108175), and cFos (Cat# sc-271243; RRID: AB_10610067) antibodies were purchased from Santa Cruz Biotechnology (Dallas, TX, USA).

Anti-GABA$_A$$\alpha$5 antibody has been validated for western blot in the website of Santa Cruz Biotechnology (https://www.scbt.com/p/gabaa-ralpha5-antibody-a-5?requestFrom=search).

4. Anti-CNTNAP4 (Cat# bs-11076R; RRID not available) and CNTNAP4 (Cat# orb544737; RRID not available) antibodies were purchased from Bioss (Beijing, China) and Biorbyt LLC (San Francisco, CA, USA), respectively.

These two antibodies have been validated for western blot and immunohistochemistry by our group in *CNTNAP4* knockout mice previously. Please refer to *Zhang W, Zhou M, Lu W, Gong J, Gao F, Li Y, Xu X, Lin Y, Zhang X, Ding L, Zhang Z, Li G, Chen X, Sun X, Zhu X, Xu P, Zhang Y. CNTNAP4 deficiency in dopaminergic neurons initiates*

parkinsonian phenotypes. Theranostics. 2020 Feb 10;10(7):3000-3021.

5. Anti-GAPDH (Cat# 60004-1; RRID: AB_2107436) antibody was purchased from Proteintech Group (Rosemont, IL, USA).

6. Anti-parvalbumin (PV, #235; RRID not available) antibody was purchased from Swant Inc. (Rte Ancienne Papeterie Marly Innovation Center, Marly, Switzerland).
PV235 antibody has been validated for immunohistochemistry and cited by 23 previously (https://www.labome.com/product/SWant/PV235.html). Please refer to *Ingold I, Berndt C, Schmitt S, Doll S, Poschmann G, Buday K, et al. Selenium Utilization by GPX4 Is Required to Prevent Hydroperoxide-Induced Ferroptosis. Cell. 2018;172:409-422.e21*

7. DyLight 488 goat anti-mouse IgG (H+L) (Cat# 70-GAM4882; RRID not available) and DyLight 594 goat anti-rabbit IgG (H+L) (Cat# 70-GAR5942; RRID not available) were purchased from Multi Sciences (Hangzhou, China).
These two antibodies have been validated for immunohistochemistry by our group previously. Please refer to *Zhang W, Zhou M, Lu W, Gong J, Gao F, Li Y, Xu X, Lin Y, Zhang X, Ding L, Zhang Z, Li G, Chen X, Sun X, Zhu X, Xu P, Zhang Y. CNTNAP4 deficiency in dopaminergic neurons initiates parkinsonian phenotypes. Theranostics. 2020 Feb 10;10(7):3000-3021.*
Also, please refer to the manufacturer's website for validation (https://www.liankebio.com/dylight-series-fluorescent-secondary-antibodies-1552.html).

8. Horseradish peroxidase (HRP)-labeled goat anti-rabbit IgG (Cat# A0208; RRID: AB_2892644) and HRP-labeled goat anti-mouse IgG (Cat# A0216; RRID: AB_2860575) were purchased from Beyotime Biotechnology (Shanghai, China).
