# Supplementary material for: Lactobacillus reuteri normalizes altered fear memory in male Cntnap4 knockout mice
Source: eBioMedicine. 2022 Nov 15;86:104323. doi: 10.1016/j.ebiom.2022.104323 (PMC9672961; doi:10.1016/j.ebiom.2022.104323)

Figure 5a

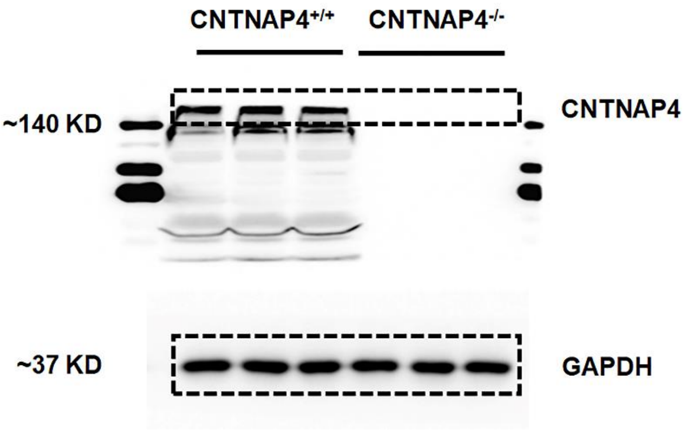

Figure 5b

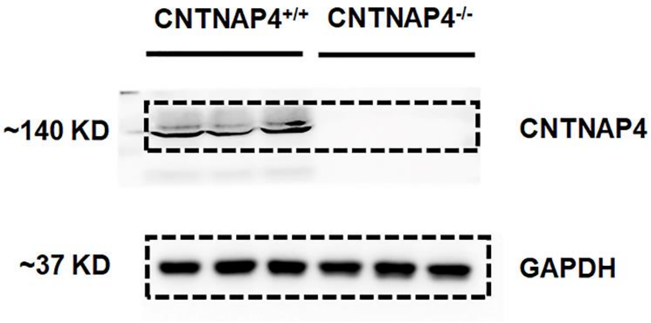

Figure 5e

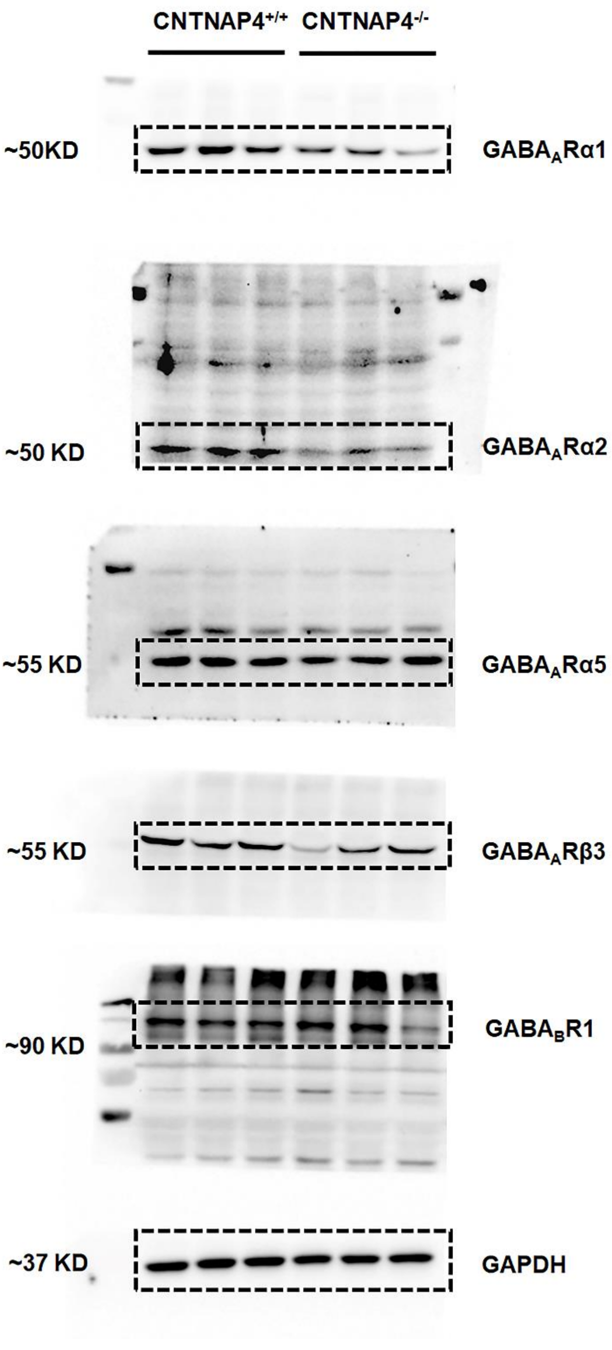
CNTNAP4+/+
CNTNAP4-/-
~50KD
GABAARα1
~50 KD
GABAARα2
~55 KD
GABAARα5
~55 KD
GABAARβ3
~90 KD
GABABR1
~37 KD
GAPDH

Figure 5f

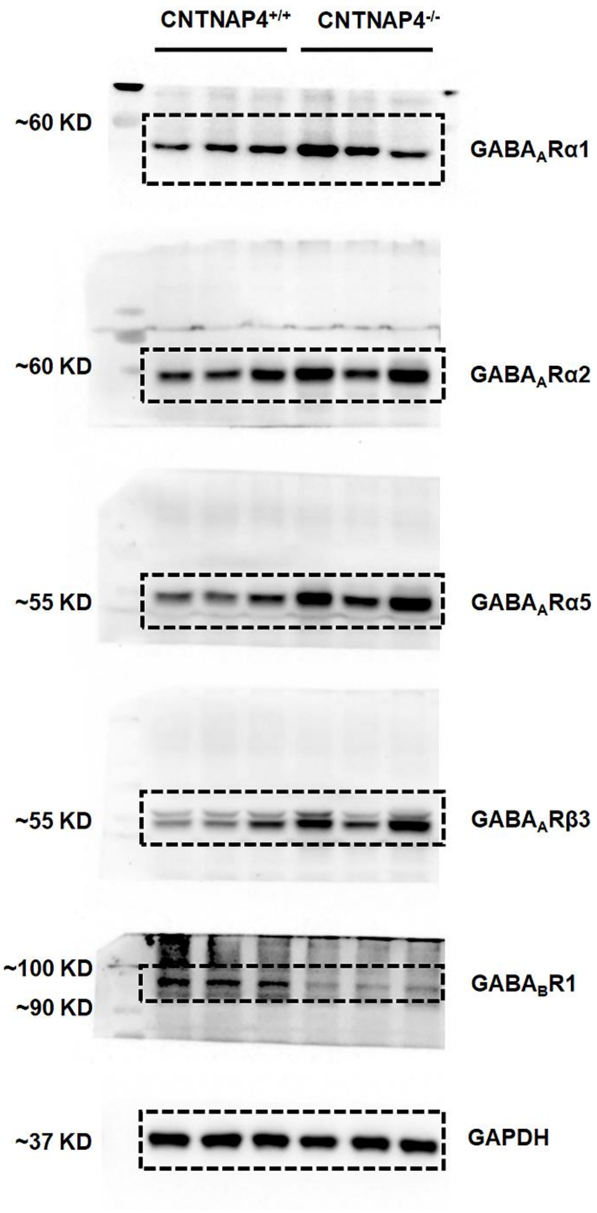

CNTNAP4+/+
CNTNAP4-/-
~60 KD
GABAARα1
~60 KD
GABAARα2
~55 KD
GABAARα5
~55 KD
GABAARβ3
~100 KD
~90 KD
GABABR1
~37 KD
GAPDH

Supplementary Figure 8a

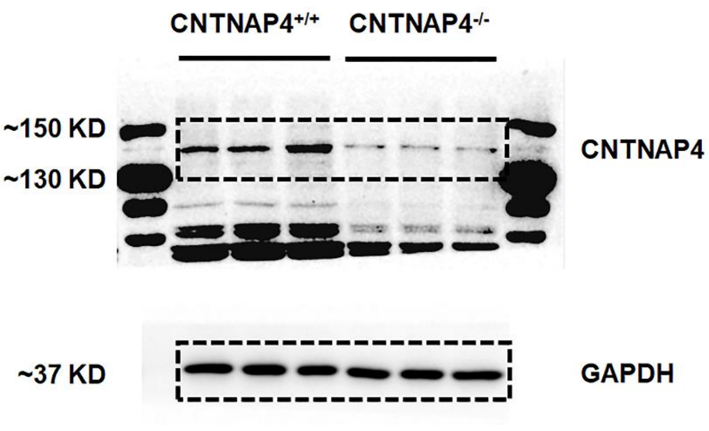

Supplementary Figure 8b

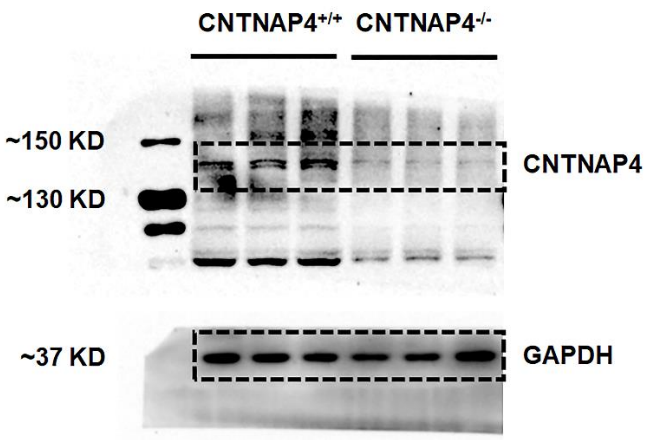

Supplementary Figure 8c

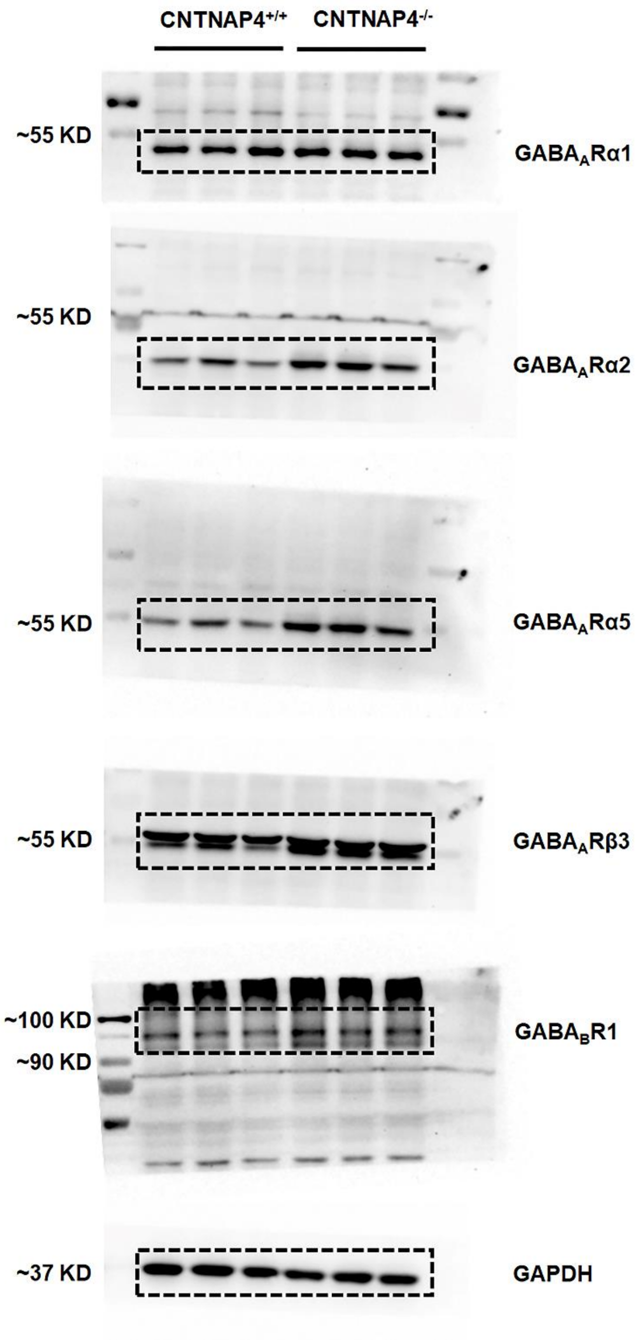

Supplementary Figure 8d

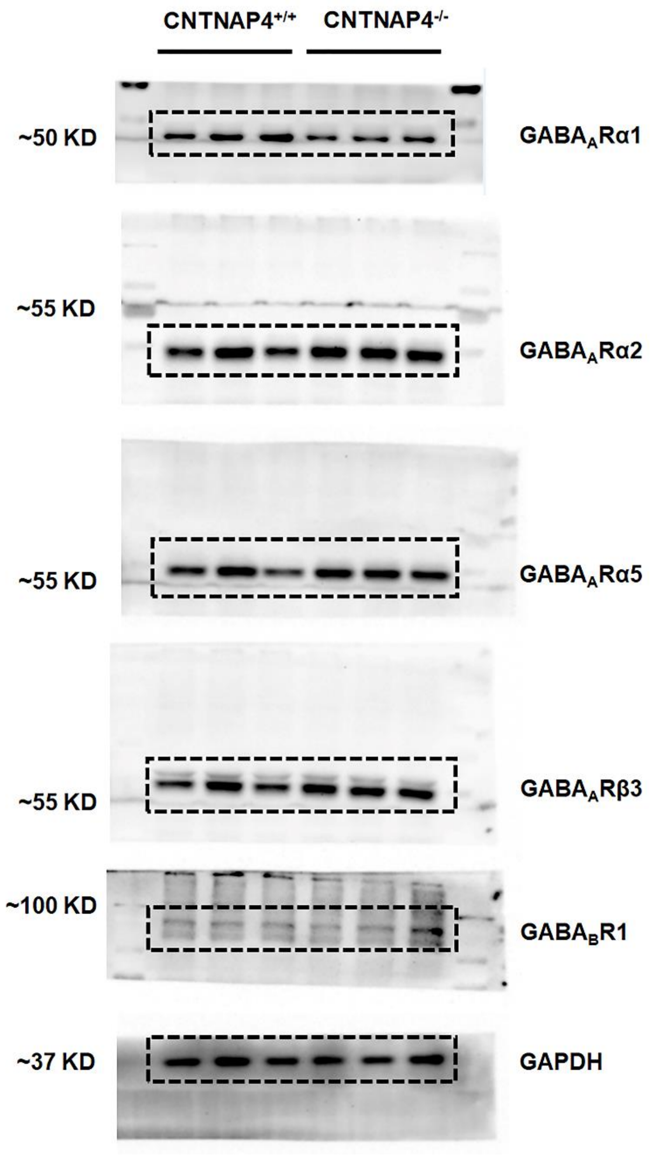

Supplement: Supplemental Western blots [file mmc4.pdf]
